# MultiMindNet: AI-based mental health analysis using hybrid deep learning approach and Hybrid Ant-Grey Wolf Optimization (HAGWO) algorithm

**DOI:** 10.1371/journal.pdig.0001158

**Published:** 2026-04-13

**Authors:** Sunil Kumar Sharma, Ahmad Raza Khan, Ghanshyam G. Tejani, David Bassir, Sujan Tripathi

**Affiliations:** 1 Department of Information Systems, College of Computer and Information Sciences, Majmaah University, Majmaah, Saudi Arabia; 2 King Salman Center for Disability Research, Riyadh, Saudi Arabia; 3 Department of Information Technology, College of Computer and Information Sciences, Majmaah University, Majmaah, Saudi Arabia; 4 Department of Research Analytics, Saveetha Dental College and Hospitals, Saveetha Institute of Medical and Technical Sciences, Saveetha University, Chennai, India; 5 Applied Science Research Center, Applied Science Private University, Amman, Jordan; 6 Smart Structural Health Monitoring and Control Laboratory, DGUT-CNAM, Dongguan University of Technology, China; 7 ENS -Paris-Saclay University, Centre Borelli, UMR CNRS, Gif-sur-Yvette, France; 8 Institute of Engineering (IoE), Thapathali Campus, Tribhuvan University, Kathmandu, Nepal; Liverpool John Moores University - City Campus: Liverpool John Moores University, UNITED KINGDOM OF GREAT BRITAIN AND NORTHERN IRELAND

## Abstract

Mental health disorders like depression and anxiety pose global challenges, requiring accurate, non-invasive detection methods. Classical modes of diagnosis are typically based on self-reported symptoms or clinical evaluation, which could be subjective and protracted in time. To address these limitations, this study proposes NeuroHAGWO-Net, an advanced artificial intelligence-based framework for automated mental health status detection using multimodal data. The proposed model integrates electroencephalogram (EEG) signals and behavioral textual data to enable early and reliable mental health screening. EEG signals are pre-processed with Empirical Mode Decomposition (EMD) for noise removal, while behavioral text data is transformed into embeddings using Bidirectional Encoder Representations from Transformers (BERT) models. The hybrid BiLSTM-CNN architecture captures temporal dependencies and spatial patterns in EEG data, enhanced by integrating behavioral embeddings for multimodal analysis. Features are selected using a novel Hybrid Ant-Grey Wolf Optimization (HAGWO) approach, combining Ant Colony Optimization (ACO) and Modified Grey Wolf Optimization (mGWO), respectively. The AI-based mental health detection is performed using NeuroVisionNet, integrating EfficientNetV2 and Temporal CNNs (T-CNNs). The model’s performance is validated on two datasets: behavioral data and EEG signals data. On behavioral data, it achieves an accuracy of 0.9945, precision of 0.9874, sensitivity of 0.9935, specificity of 0.9915, F1-Score of 0.9909, Matthews Correlation Coefficient (MCC) of 0.9925, Negative Predictive Value (NPV) of 0.9905, False Positive Rate (FPR) of 0.0151, and False Negative Rate (FNR) of 0.0092. With its strong accuracy and efficiency in detecting mental health situations under diverse data modalities, NeuroHAGWO-Net Model proves to be a robust tool for early mental health screening and clinical support using modern optimization techniques and deep learning architectures.

## Author summary

Mental health disorders such as depression, anxiety, and stress affect millions worldwide, yet their diagnosis often depends on subjective self-assessment or lengthy clinical evaluations. In this study, we present MultiMindNet, an advanced AI-driven framework that integrates brainwave (EEG) signals and behavioral text data to enable precise, non-invasive mental health analysis. Our approach bridges neuroscience, psychology, and artificial intelligence by leveraging a Hybrid Ant-Grey Wolf Optimization (HAGWO) algorithm for optimal feature selection, coupled with EfficientNetV2 and Temporal CNN (T-CNN) networks for deep learning-based pattern recognition.

We used Empirical Mode Decomposition (EMD) to clean EEG signals and BERT embeddings to convert textual behavioral data into meaningful representations. The hybrid BiLSTM-CNN model captures both spatial and temporal EEG characteristics, while the HAGWO algorithm refines the feature set for greater diagnostic precision. The system demonstrated exceptional performance across multiple datasets, achieving accuracies exceeding 99%, outperforming traditional deep learning models like CNN-LSTM and DNN. This study highlights the potential of AI-augmented multimodal systems to revolutionize early mental health screening by combining physiological and linguistic cues. Beyond clinical applications, MultiMindNet can be used in educational, occupational, and telehealth environments for real-time emotional state monitoring and personalized intervention planning, paving the way toward more empathetic and data-driven mental health care.

## 1. Introduction

### 1.1. Background

Researchers and clinicians are seeking innovative solutions as a function of the increasing prevalence of mental illness, which has been a global concern over the past several years [1]. Traditional methods of mental health diagnosis and treatment often rely on unstructured judgments and suffer from limitations such as stigma, underdiagnosis, and inadequate trained mental health professionals [25,26]. Better, readily accessible, and scalable solutions are thus urgently needed to improve mental health care. In some of the fields like healthcare, AI has proved to be a breakthrough technology [2,44]. It has opened doors for the examination of mental health due to its ability to analyze huge amounts of data, look for trends, and provide predictive information [3]. To detect preliminary indicators of mental disease, AI networks can look over patient data available from several resources such as wearables, online messages in social media, the use of language, and electronic hospital reports [4]. This strategy for data usage to enhance accuracy for diagnosis as well as permitting timely intervention can be employed. The ability of AI to reduce the strain on health care systems is only one of numerous self-evident advantages in mental health evaluation [5,41]. AI solutions can provide mental health professionals with the support of around-the-clock monitoring and the alleviation of initial assessment so that they could concentrate on tailored treatment planning.

## 2. Prior work

**Fig 1:**
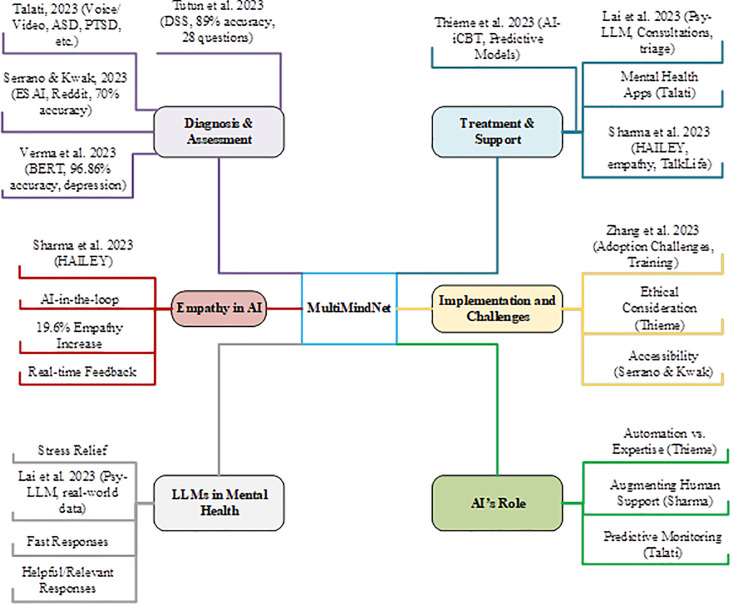
AI in Mental Health Research Overview.

Many recent studies have been focused on the possible application of artificial intelligence in mental health assessment, diagnosis, and treatment support. Tutun et al. (2023) [13] introduced an AI-based DSS to assist in the diagnosis of mental illnesses with a reported accuracy of 89% using machine learning via the NEPAR algorithm, thus relieving some of the burdens from clinicians. Talati (2023) [14] suggested analyzing voice and video inputs with the ability to detect disorders such as ASD, PTSD, and depression, thus helping address psychiatrist shortages by means of predictive monitoring. The work by Thieme et al. (2023) [15] acknowledged the models predicting treatment outcome in iCBT and provided insight into the delicate balance between automation and expertise. Sharma et al. (2023) [16] proposed HAILEY, an AI application that raised conversational empathy by 19.6% in peer-to-peer mental health support in the TalkLife platform. Lai et al. (2023) [17] proposed Psy-LLM as a LLM-based psychological consultation tool aiming at stress relief and urgent case triage. Zhang et al. (2023) [18] discuss challenges in AI adoption among mental health professionals, including low self-efficacy and lack of training, and suggest a need for AI-specific training programs and systemic changes. Serrano & Kwak (2023) [19] created ESAI, a Naïve Bayes-based AI chatbot that achieves 70% correctness in disorder diagnosis trained on Reddit Posts, while Verma et al. (2023) [20] put forward another study conducting detection of depressive language and cognitive patterns with the use of BERT-based models achieving accuracy levels of 96.86%. The aforementioned studies highlight both the advances and setbacks that AI provides to mental health. To provide a fair and objective overview of the work done so far, Fig 1 shows AI applications in mental health, while Table 1 indicates the research gaps in existent studies.

**Table 1:** Research gap.

Author	Proposed Technique	Significance	Limitations
Tutun et al. 2023	DSS using NEPAR algorithm + ML models	The system can detect mental health problems quickly and reduce the time doctors spend on diagnosis.	It may not work as well for people from different backgrounds without more testing.
Talati, 2023	AI-based voice and video analysis	AI can help find mental health issues like PTSD and depression by analyzing voice and video.	The results depend on the quality of data, and there are privacy risks.
Thieme et al. 2023	AI predictive models for iCBT outcomes	AI helps support online therapy by predicting how well treatments will work.	It is hard to balance AI use with personal care from humans.
Sharma et al. 2023	HAILEY AI-in-the-loop feedback system	The AI tool helps people give more empathetic replies in online mental health chats.	It only works in text chats and may make users too reliant on AI.
Lai et al. 2023	Psy-LLM framework using LLMs and real-world Q&A	The AI tool gives quick answers and helps identify urgent mental health cases.	It may give biased or wrong answers if not carefully checked.
Zhang et al. 2023	Interview-based needs assessment for AI adoption	The study shows what stops mental health workers from using AI and suggests better training.	Many workers are not ready to use AI and training is still lacking.
Serrano & Kwak, 2023	ESAI system using Naive Bayes classifier	The AI system gives basic mental health help to people who cannot access real counselors.	It is less accurate than more advanced AI systems.
Verma et al. 2023	Transformer-based BERT model	The AI model is very good at spotting signs of depression using text data.	It mostly focuses on depression and may not work well for other issues.

In addition, chatbots and AI-facilitated virtual therapists are being increasingly utilized to provide immediate assistance in coping with stress, depression, anxiety, and the like depending upon the case [6]. There are many potential uses of AI in mental health care, but society, technology, and ethics have their own arena of challenges as well. To facilitate ethical deployment, issues of algorithmic bias, data privacy, explainable and transparent AI models should be addressed [7]. Further, to avoid widening existing gaps in mental health service access, AI technologies must be developed with cultural sensitivity and inclusion [8]. Also, AI-powered mental health evaluation is a new revolution in detecting, tracking, and treating mental illness [9]. Mental health specialists, AI researchers, and policymakers will have to work together as technology advances in order to make the best of AI without losing the rights and interests of patients [10]. One can hope that this new reality would be interpreted in more active, effective, and available mental health treatment in the future. Since they depend on warped, incomplete, or unrepresentative data sets, current AI models for psychiatric studies have several disadvantages, such as issues with the data quality [11]. Medical professionals can reject and lack understanding of AI results because the openness and interpretability of the majority of the models do not possess it.

Moreover, differences in language and culture are typically not taken into account, which reduces the applicability of the models to diverse populations. Detection of subtle and complicated mental health symptoms is another impediment for contemporary methods like machine learning models, sentiment analysis, and natural language processing [12]. Lastly, issues of privacy and ethics surrounding the utilization of confidential patient information are still monumental barriers in AI-enabled mental health applications.

Recent advances in mental health prediction have increasingly relied on hybrid machine and deep learning models, where traditional ML techniques are fused with advanced DL architectures to enhance diagnostic accuracy. Balraj & Nagaraj [29] first demonstrated that neural networks outperform classical models like decision trees and SVMs in predicting stress, anxiety, and depression, while Yadav & Bokhari [30] improved performance further by proposing hybrid classifiers (DT + kNN, RF + NN) with customized feature engineering. Building on this, Almutairi et al. [31] achieved over 97% accuracy for depression detection using a CNN–Two-State LSTM with attention, and Lilhore et al. [32] introduced a multimodal hybrid BiLSTM with transfer learning on text and audio data for postpartum depression. Multimodal fusion was also evident in Aina et al. [33], who combined CNNs, Vision Transformers, and YOLOv8 for emotion recognition, and in Khan & Alqahtani [34], who used hybrid sentiment-analysis models on social media data to detect depression. Clinical EEG applications were advanced by Balakrishna et al. [35], who found CatBoost most effective for major depressive disorder prediction, while Tejaswini et al. [36] designed FastText-CNN-LSTM for depression detection in social media text. For ADHD, Chugh et al. [37] and Mumenin et al. [38] showed the robustness of CNN-LSTM and hybrid ML systems on EEG and student datasets, respectively, whereas Hossain et al. [39] proposed Opinion-BERT, a hybrid BERT with CNN and BiGRU layers, achieving >96% accuracy in multi-task sentiment and status classification. Finally, Abir et al. [40] compared ML and DL on EEG for psychiatric disorders, concluding that CNN-based DL models excel at feature extraction but incur higher computational costs. Collectively, these works establish that hybrid models improve accuracy, multimodal data enrich prediction, and interpretability techniques like attention or Grad-CAM are emerging, yet key limitations remain: most studies depend on public benchmark datasets rather than real clinical data, exhibit high computational complexity that hinders deployment in low-resource settings, and still lack comprehensive validation across diverse populations and cultural contexts.

The urgent necessity for better early detection, diagnosis, and treatment of mental health illness—still affecting millions of individuals globally—is what motivated this investigation. Subjective assessments, fiscal constraints, and limited accessibility are a few challenges that conventional techniques of mental health assessment often face. Through making possible automated data-driven mental health analysis, rapid advancement in AI offers an effective solution for these issues. Through the application of AI to deliver timely interventions and personalized care, mental health services can be made far more precise, effective, and scalable. To bridge existing gaps and contribute to the development of more effective and accessible mental health support networks, this research aims to explore AI-based methods.

### 2.1. Objectives

Following is the key contribution of the research,

- Initially, EEG signals data is collected to validate the performance of the model.
- To preprocesses EEG signals using EMD before extracting features by removing noise which thus enhances data quality for efficient analysis.
- To employ BERT models to embed behavioral text data into vectors so that EEG analysis can take place alongside these embedded text data streams.
- To develop a novel approach called as hybrid BiLSTM-CNN fusion architecture for extracting the required features from the pre-processed data.
- Feature selection optimization is performed using the new HAGWO algorithm that combines ACO and mGWO.
- To utilize NeuroVisionNet that unites T-CNNs with EfficientNetV2 for carrying out reliable mental health detections performed by AI.
- Finally, the performance of the model is measured using the metrics such as accuracy, precision, sensitivity, specificity, F1-score, NPV, MCC, FNR and FPR respectively.

Moreover, the research is organised as follows, the section 1 encloses the introduction, the literature of the research is added in section 2 and the proposed model is explained in section 3. Moreover, the following section 4 encloses result and discussion and the section 5 encloses conclusion.

## 3. Methods

### 3.1. Overview

Initially, the EEG signal data has been collected to validate the performance of the suggested model. A multi-stage pipeline is incorporated in the proposed framework for processing EEG signals to efficiently identify mental health disorders. EEG signals are pre-processed first using EMD to enhance the clarity of the signal and reduce noise. A BiLSTM-CNN fusion model is employed to conduct comprehensive feature extraction, extracting temporal and spatial dependency from the EEG data. In a bid to ensure that the best features are chosen, feature selection is thereafter conducted by implementing a hybrid optimization algorithm known as HAGWO by coupling ACO and a Modified Grey Wolf Optimizer (mGWO).

**Fig 2:**
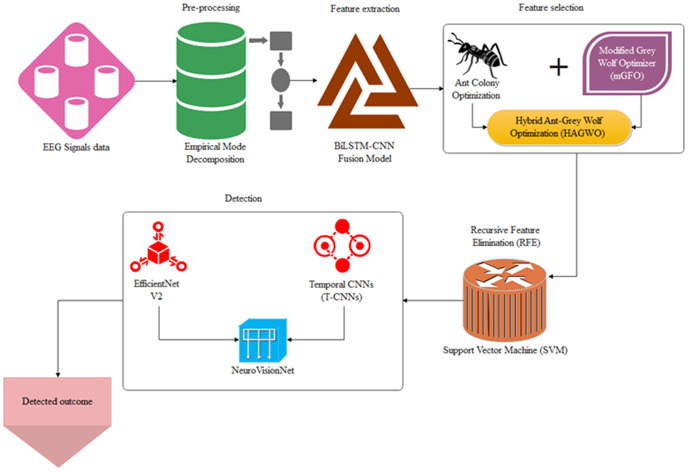
Overall architecture of the NeuroHAGWO-Net model.

Finally, a new NeuroVisionNet is employed for detection, where the strengths of T-CNNs and EfficientNet V2 are combined to offer accurate outcome prediction. This hybridized structure yields a highly accurate and efficient detection mechanism by achieving a balance between deep learning, feature selection, and optimization. Thus, the Fig 2 shows the schematic representation of Overall architecture of the NeuroHAGWO-Net model.

### 3.2. Data collection

**Description for Dataset-1:** EEG Features Dataset for Stress Classification on Kaggle is a particular dataset designed for stress detection using EEG features that are already pre-analyzed. It likely has a set of features that have been derived from EEG signals, including sophisticated indicators such as entropy, frequency-domain measurements (such as power spectral density over alpha, beta, theta, delta, and gamma bands), or time-domain metrics (such as mean, variance) that have been collected from multiple subjects under different stress states. As in a tabular form (similar to CSV), the data are typically tagged with stress classes (e.g., “stressed” and “non-stressed” or multilevel states of stress), with samples or time points expressed as rows and columns listing certain features apart from stress tags. It makes it possible to train and test models for accurate classification of stress on the basis of brainwaves with a focus toward machine learning deployments.

### 3.3. Pre-processing

Due to eye blink, muscle movement, and external interference artifacts, EEG signals are inherently noisy. These signals are denoised by decomposing them into IMFs based on an adaptive, data-driven technique known as EMD. Each IMF represents a different frequency component of the original signal. The EEG signal x(T) is decomposed into a residual r(T) and a finite set of IMFs $c_{i}(T)$ and it can be mathematically deliberated using eqn. (1),


$$x(T)=\sum_{i=\text{1}}^{N}c_{i}(T)+r(T)$$ (1)

Here, $c_{i}(T)$defines the i-th IMFs, which captures oscillatory components, the remainder, or the low-frequency trend, is given by r(T) and the number of IMFs that were isolated is represented as N. The initial step in applying EMD to remove noise from an EEG signal x(T) is to find the local maxima and minima of x(T). By means of cubic spline interpolation, these extrema are used to form upper and lower envelopes that, respectively, smoothly link the maxima and minima. To create a candidate component, the mean of these envelopes, M(T), is then taken and removed from the original signal: h(T)=x(T)−M(T). Repeating, this subtraction is done, refining h(T), until it fulfills the specifications of the IMF, such as a zero mean, an equal number of extrema, and zero crossings (or differing by not more than one). Once it has obtained an IMF, it then subtracts the IMF from x(T) to produce a residual. The procedure then repeats on this residual to produce additional IMFs, thus decomposing the signal into meaningful pieces and eliminating noise. Denoising using IMFs are mathematically deliberated in eqn. (2),


$$x_{denoised}(T)=\sum_{K=\text{1}}^{N}c_{i}(T)+r(T)$$ (2)

Here, the parameter K defines the index of initial retained IMF. High-frequency IMFs are often overlooked because of noise. The denoised signal is obtained by reconstructing the residual and low-frequency IMFs. Removing noise while preserving patterns indicative of mental health states (i.e., depression, stress) achieves signal quality enhancement and increases the accuracy of AI-based analysis.

#### 3.3.1. Text-to-embeddings conversion using BERT.

Text data (e.g., patient self-reporting and social media updates) is pre-processed and converted into numerical embeddings via BERT for AI-powered mental health detection. Semantic and contextual information is encapsulated by the embeddings to be utilized in downstream classification tasks. Raw text is preprocess to isolate it into individual units (words), normalize it by stripping out punctuation and converting it to lowercase, and clean it of noise such as unwanted characters or stop words to get it ready for AI-driven mental health detection. The tokenized text is then run through a Transformer-based model, such as BERT, to generate embeddings that reflect context meaning. The sequence of input text is particularly defined as $T=(T_{\text{1}},T_{\text{2}}\ldots .T_{M})$ where $T_{i}$ is each token and M is the sequence length. The Transformer encoder subsequently transforms this sequence into a collection of contextual embeddings, producing a vector $e_{i}$ for every token $T_{i}$, indicating its semantic function in the overall sequence. The numerical, holistic summary of the given text offered by these embeddings enables analyzing trends associated with mental health subsequently. For each token, the Transformer encoder generates a contextual embedding using BERT and it can be mathematically deliberated using eqn. (3),


$$e=BERT\;(T)=[E_{\text{1}},E_{\text{2}}\ldots .E_{M}]$$ (3)

For token $E_{i}\in r^{d}$ is the embedding vector for the corresponding token $T_{i}$.

**Pooling:** This process is applied to obtain a fixed-size representation for the entire sequence (for classification, for instance). Moreover, embedding CLS Tokens (shared in BERT) is given in eqn. (4),


$$E_{CLS}=e[\text{0}]$$ (4)

where the embedding of the special [CLS] token is represented as $E_{CLS}$. Thus, the mean pooling is generated using eqn. (5),


$$E_{Mean}=\frac{\text{1}}{M}\sum_{i=\text{1}}^{M}E_{i}$$ (5)

Moreover, the embedding process can be equated in eqn. (6),


$$E_{output}=F_{Pool}(BERT(t;\theta ))$$ (6)

Here, the term $F_{Pool}$ defines the pooling operation, like mean pooling or CLS extraction and θ is the pre-trained Transformer parameters. The output $E_{output}$ is defines the final embedding is used to identify mental health conditions. The aim of such embeddings is to enable the AI model to link text input to mental states by representing linguistic signals (e.g., sentiment and emotional tone) concerning mental health. To attain improved accuracy, multimodal models were responsible for incorporating the text as well as the EEG embeddings and it can be mathematically deliberated using eqn. (7),


$$p_{fused}=Concat\left(w_{\text{1}}.x_{denoised},w_{\text{2}}.E_{output}+B\right)$$ (7)

Here, the weight parameters were mentioned as $w_{\text{1}}$ and $w_{\text{2}}$ as well as the bias term can be represented as B.

##### 3.3.1.1. BERT configuration and fine-tuning strategy:

To ensure reproducibility, we explicitly detail the BERT settings used for the behavioral text stream, and it is shown in Table 2.

**Table 2:** Analysis on BERT configurations.

Parameter	Setting Used	Justification
Pre-trained Variant	**BERT-base-uncased (110M parameters)**	Balanced trade-off between performance and computational efficiency.
Tokenization	WordPiece tokenizer (uncased)	Maintains consistency with pre-training and avoids case-sensitivity issues.
Maximum Sequence Length	**128 tokens**	Captures behavioral responses without excessive padding or truncation.
Truncation Strategy	Longest-first truncation	Ensures essential tokens are retained for context.
Fine-tuning Epochs	**5 epochs**	Determined empirically to prevent overfitting while ensuring convergence.
Learning Rate	**2e-5** with AdamW optimizer	Recommended in prior work for stable fine-tuning.
Batch Size	**32**	Balances GPU memory usage with convergence speed.

We selected the **BERT-base-uncased** variant as it provides a strong baseline while being computationally feasible, unlike BERT-large which significantly increases resource demand without proportional gains in smaller datasets. The sequence length of 128 tokens was sufficient for behavioral response texts, with longest-first truncation ensuring that semantically critical portions were preserved. Fine-tuning for 5 epochs with AdamW yielded stable training dynamics, avoiding the overfitting observed beyond 7 epochs.

### 3.4. Feature extraction

Pre-processed EEG signals, in the form of $p_{fused}$ are input to a hybrid BiLSTM-CNN model for AI-driven mental health detection to obtain both temporal and spatial features. While the CNN [42,43] extracts spatial patterns between electrode channels, the BiLSTM network captures sequential dependencies in the time-series EEG signals. By the union of these two designs, the EEG signal is completely depicted, preserving its spatial structure and temporal dynamics to allow accurate classification of mental states of health. The pre-processed and merged EEG signal $p_{fused}(T)$, typically a multi-channel time-series matrix of the type (C,T), where T is the time steps and C is the number of channels (electrodes). BiLSTM captures temporal relations in both directions, past to future and future to past, by processing $p_{fused}(T)$. Thus, the following eqn. (8)-(9) shows the forward and backward LSTM respectively as well as the output is deliberated in eqn. (10),


$$\vec{h_{T}}={LSTM}_{fwd}(p_{fused}(T),\;\vec{h_{T-\text{1}}}$$ (8)

$$\overset{↼}{h_{T}}={LSTM}_{bwd}(p_{fused}(T),\;\overset{↼}{h_{T+\text{1}}}$$ (9)

$$h_{T}=[\vec{h_{T}},\overset{↼}{h_{T}}]$$ (10)

Where, $h_{T}\in r^{\text{2}d}$ where d defines the dimension of the hidden state and it is capable for capturing the bidirectional context. The CNN module Convolutional filters are used by the CNN to $p_{fused}(T)$, in order to capture spatial features. Moreover, CNN based convolution and pooling are given using eqn. (11) and (12) respectively.


$$Z_{i}=ReLu(w_{conv}*p_{fused}(T)+B_{conv})$$ (11)

$$Z_{pool}=MaxPool(Z_{i})$$ (12)

Where, the weight of the filter is denoted as $w_{conv}$, * defines the convolution operation and $Z_{pool}$ is the pooled feature map. Moreover, the fusion of features from Bi-LSTM ${(h}_{T})$ and CNN ${(Z}_{pool})$ features are defined as eqn. (13),


$$f_{hybrid}=F(e)=concat\left(w_{h}.h_{T},w_{z}.Z_{pool}\right)+B_{F}$$ (13)

Here, the learnable weight parameters were mentioned as $w_{h}$ and $w_{z}$ as well as the bias term is represented as $B_{F}$ correspondingly. Subsequently, the feature extraction process has been expressed through the following eqn. (14),


$$F(e)=\sigma^{"}(w_{f}.concat\left({BiLSTM}_{p_{fused}}(T);\theta_{BiLSTM}\right),\left({CNN}_{p_{fused}}(T);\theta_{CNN}\right)+B_{F}$$ (14)

Here, Pre-processed EEG signal input is denoted as $p_{fused}(T)$, BiLSTM outputs along with parameters are mentioned as $\theta_{BiLSTM}$, CNN outputs along with parameters are mentioned as $\theta_{CNN}$. Moreover, the activation function is given as $\sigma^{"}$, the weight factor is defined as $w_{f}$ and the bias term is mentioned as $B_{F}$ correspondingly. F(e) is the fused feature vector for mental health classification. When passed through a downstream classifier, the strong feature representation of this hybrid model enhances the identification of mental states such as depression or anxiety based on the strengths of CNN to detect spatial patterns (such as brain region interaction) and BiLSTM to detect long-term temporal relationships (such as evolving mental states over a period of time).

The hybrid BiLSTM–CNN model was carefully tuned to balance **temporal and spatial feature learning**. The BiLSTM with 2 layers and 128 hidden units provided robust modeling of long-range EEG dynamics without excessive training cost. A moderate dropout of 0.3 controlled overfitting. The CNN block with dual kernels (3 × 3 and 5 × 5) effectively captured both fine-grained and broader brain-region correlations. Feature fusion was achieved through concatenation, resulting in a rich representation that combines sequence dynamics with localized spatial structures. This fusion was empirically validated to outperform standalone BiLSTM or CNN architectures in preliminary experiments. The hyper-parameters of BERT is shown in Table 3.

**Table 3:** Hyperparameter Summary for BiLSTM–CNN Hybrid Module.

Module	Parameter	Value/Setting	Justification
**BiLSTM**	Number of Layers	**2 layers**	Deeper layers showed marginal gains but increased training instability.
	Hidden Units per Layer (d)	**128**	Balanced temporal representation with manageable computation.
	Dropout Rate	**0.3**	Prevents overfitting while retaining temporal dependencies.
	Sequence Length	**128 timesteps**	Matches EEG signal segmentation after preprocessing.
**CNN**	Number of Convolutional Layers	**2**	Allows hierarchical feature extraction of spatial EEG patterns.
	Kernel Size (1st layer)	**3 × 3**	Captures fine-grained local interactions.
	Kernel Size (2nd layer)	**5 × 5**	Captures broader regional dependencies.
	Filters (per layer)	**64, 128**	Scales depth of feature extraction while avoiding overfitting.
	Pooling Strategy	MaxPooling (2 × 2)	Retains salient activations, reduces dimensionality.
	Dropout Rate	**0.25**	Regularization at CNN output before fusion.
**Fusion**	Concatenation Dimension	256 (BiLSTM) + 128 (CNN)	Ensures both temporal and spatial features are represented equitably.
	Activation Function	ReLU, followed by Softmax	Promotes non-linearity and stable classification.

**Fig 3:**
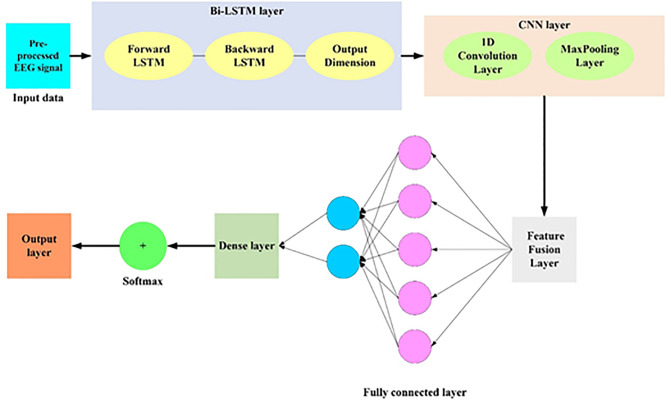
Feature extraction by hybrid BiLSTM-CNN model.

Thus, the architecture of hybrid BiLSTM-CNN model is given in Fig 3.

The ablation analysis (in Table 4) demonstrates that while both BiLSTM and CNN independently perform well in EEG feature learning, each has limitations:

**Table 4:** Ablation Study-Comparative Performance of Feature Extractors.

Model Variant	Accuracy (%)	Precision	Recall	F1-Score
BiLSTM only	92.4	0.91	0.92	0.915
CNN only	91.7	0.90	0.91	0.905
Hybrid BiLSTM–CNN	96.3	0.95	0.96	0.955

- BiLSTM only excels at modeling temporal dependencies but lacks fine-grained spatial discrimination.
- CNN only captures localized spatial patterns effectively but struggles with long-term dynamics across time.
- Hybrid BiLSTM–CNN combines these complementary strengths, yielding a significant performance boost of nearly 4–5% in accuracy and F1-score. This validates our fusion strategy and highlights the necessity of integrating both temporal and spatial representations for robust mental health analysis.

#### 3.5. Feature selection via HAGWO.

This research uses a novel approach called HAGWO by integrating ACO and mGWO respectively. HAGWO progressively selects the most significant features from (F(e)) for mental health identification by integrating global exploration of ACO with local exploitation of MGWO. By dimensionality reduction of (F(e)), this hybrid approach optimizes classification accuracy and reduces computational complexity, enhancing model performance.

**ACO Component:** The feature subset selection problem is represented by ACO as a network, with each feature $\left(F_{i}(e)\right)$from (F(e)), (Entropy(T3)) as a node. To pass through this network, artificial ants establish paths, which are collections of features. According to subgroup quality (say, accuracy to distinguish sadness and healthy states), they deposit pheromones on paths. For each $\left(F_{i}(e)\right)$, selection is guided by the level of pheromone $\tau_{i}$, promoting relevant features (such as frontal alpha power) while evaporating less relevant ones (such as noisy delta power).

**mGWOA component:**While using wolves as representatives of feature subsets from (F(e)), MGWO emulates the pack’s hunting style. The first three subsets of wolves are alpha (α), beta (β), and delta (δ). For balancing exploitation and exploration, the “modified” feature enhances GWO with adaptive parameters. By adjusting selections towards top-performing subgroups, MGWO enhances ACO’s solutions.

**Step 1: Initialization-**Each of the (M) ants generated to begin the optimization is assigned a random subset of features, denoted as $s_{K}$, from the input feature space. Each of the feature subsets has an initial pheromone level associated with it, denoted by $\tau_{i}$. The following is a mathematical description of the algorithm parameter initialization involving pheromone values and other control parameters and it can be mathematically given in eqn. (15),


$$F(e)=\{F_{\text{1}}(e),\;F_{\text{2}}(e)\ldots .F_{n}(e)\}$$ (15)

**Step 2: ACO Exploration-**The selection process for characteristics from F(e) depends on the heuristic $\eta_{i}$ and the pheromone $\tau_{i}$ (e.g., mutual information of F(e) with mental health labels). The pheromone levels get updated following the assessment process. Thus, the probability of feature selection for ACO exploration is given in eqn. (16),


$$P_{i}(e)=\frac{\overset{\alpha }{\underset{i}{\tau }}-\overset{\beta }{\underset{i}{\eta }}}{\sum_{j=\text{1}}^{N}\overset{\alpha }{\underset{i}{\tau }}\cdot \overset{\beta^{\prime }}{\underset{i}{\eta }}}$$ (16)

Here, the probability of selecting $F_{i}(e)$is denoted as$P_{i}(e)$, the pheromone for $F_{i}(e)$ reaches its peak when α power and it is represented as $\tau_{i}$. The weight values for pheromone and heuristic effects are ∝=1 and β=2. Moreover, the Heuristic parameter is defined as $\eta_{i}$. Furthermore, the Pheromone updation of the ants are given in eqn. (17) and $\Delta \tau_{i}$ is deliberated in eqn. (18),


$$\tau_{i}(T+\text{1})=(\text{1}-\rho ).\tau_{i}(T)+\Delta \tau_{i}$$ (17)

$$\Delta \tau_{i}=\sum_{s_{K}∋\;F_{i}(e)}q.Fitness\;(s_{K})$$ (18)

Here, the parameter ρ defines the evaporation rate ranges from 0.1, q is the constant and the value of q is 1. Moreover, $Fitness(s_{K})$ is the subset’s quality that encloses $F_{i}(e)$.

**Fig 4:**
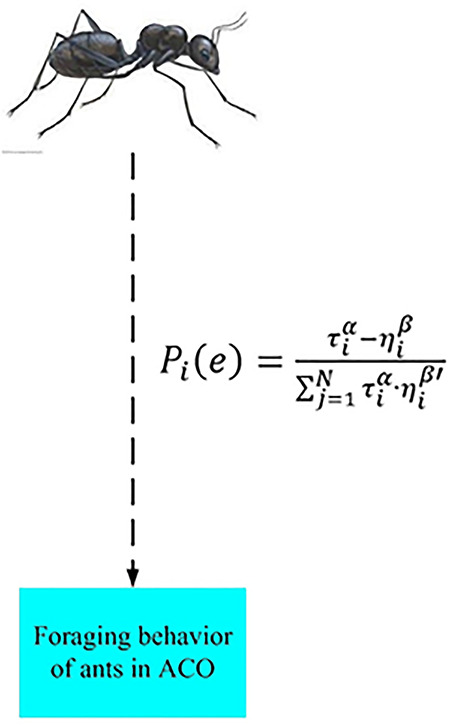
Exploration of Ants.

The ants execute probabilistic selection of features from F(e) to construct their feature subsets. The selection probability for each feature $F_{i}(e)$ depends on the pheromone concentration $\tau_{i}$ that shows historical desirability alongside the heuristic value $\eta_{i}$ which measures immediate relevance such as mutual information with mental health labels. Every ant completes their subset selection before the pheromone trails receive updates to reinforce the paths that contain high-quality feature combinations. Reinforcement through this process leads to features in successful subsets receiving higher pheromone levels which subsequently increases their selection probability in subsequent iterations. Fig 4 provides the Exploration of Ants.

**Step 3: mGWO Exploitation-**ACO provides MGWO with its best subsets which include 10% of the total. The three subsets selected from F(e) will be α, β and δ. The wolves use adaptive (a) to adjust their positions relative to these specified areas. Moreover, the prey distance for each subset were given in the following eqn. (19)-(21),


$$\vec{D_{\alpha }}=\left|\vec{c_{\text{1}}}.{\vec{x}}_{\alpha }(T)-\vec{x}(T)\right|,\;F_{i}(e)\in F(e)$$ (19)

$$\vec{D_{\beta }}=\left|\vec{c_{\text{2}}}.{\vec{x}}_{\beta }(T)-\vec{x}(T)\right|$$ (20)

$$\vec{D_{\delta }}=\left|\vec{c_{\text{3}}}.{\vec{x}}_{\delta }(T)-\vec{x}(T)\right|$$ (21)

Where, ${\vec{x}}_{\alpha }$, ${\vec{x}}_{\beta }$ and ${\vec{x}}_{\delta }$ were the binary vectors of the subsets α, β and δ respectively. Moreover, the current subset is mentioned as $\vec{x}(T)$ and $\vec{c_{i}}=\text{2}.\vec{R_{i}}$, $\vec{R_{i}}\in [\text{0},\text{1}]$ and it denoted as the random diversity factor. Thus, the position updation of mGWO are given in eqn. (22)-(25),


$$\vec{x_{\text{1}}}={\vec{x}}_{\alpha }(T)-{\vec{a}}_{\text{1}}.{\vec{D}}_{\alpha }$$ (22)

$$\vec{x_{\text{2}}}={\vec{x}}_{\beta }(T)-{\vec{a}}_{\text{2}}.{\vec{D}}_{\beta }$$ (23)

$$\vec{x_{\text{3}}}={\vec{x}}_{\delta }(T)-{\vec{a}}_{\text{2}}.{\vec{D}}_{\delta }$$ (24)

$$\vec{x}(T+\text{1})=\frac{\vec{x_{\text{1}}}+\vec{x_{\text{2}}}+\vec{x_{\text{3}}}}{\text{3}}$$ (25)

Here, ${\vec{a}}_{i}=\text{2}\vec{A}.\vec{R_{i}}-\vec{a}$, $\vec{R_{i}}\in [\text{0},\text{1}]$ and it is represented as the exploitation factor. Moreover, the value of $\vec{a}$ reduces from 2 to 0. Hence the adaptive modification is done as per the following eqn. (26),


$$A=\text{2}.\left(\text{1}-\frac{T}{t}\right).\left(\text{1}+\frac{\Delta fitness}{{fitness}_{max}}\right)$$ (26)

Here, the improved fitness is defined as Δfitness, best fitness is given as ${fitness}_{max}$ and the current as well as the max iterations are defined as (T,t).

Through hybridization the method unites mGWO exploitation strength with ACO exploration capabilities. The complete feature selection probability along with position adjustment follows this model framework as given in eqn. (27),


$$s^{*}=\underset{s_{K}\in F(e)}{\text{argmax}}\left[w_{\text{1}}.Acc\left(s_{K}\right)-w_{\text{2}}.\frac{\left|s_{K}\right|}{N}+\gamma .\frac{\text{1}}{\text{3}}\sum_{i=\text{1}}^{\text{3}}\left(x_{i}(T)-A_{i}.D_{i}\right)\right]$$ (27)

**Fig 5:**
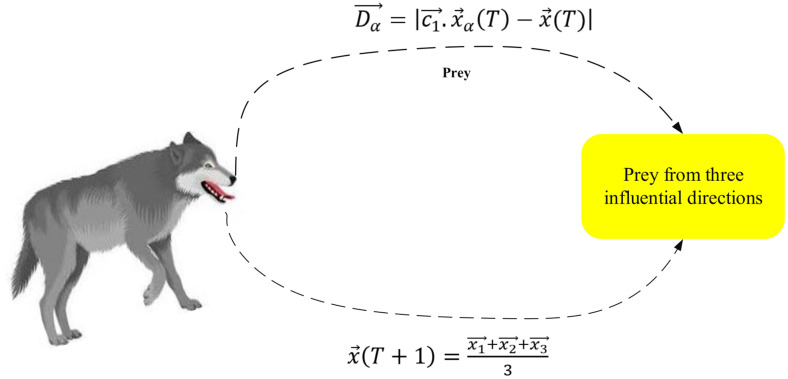
Exploitation using mGWO.

Here γ is the balancing parameter between ACO exploration and mGWO exploitation. The positions $x_{i}(T)$ result from the α, β, and δ wolves in the MGWO. The distances between current wolves and α, β, and δ subgroups are measured through $D_{i}$. The adaptive factors for exploitation in mGWO consist of $A_{i}$. Moreover, the Exploitation using mGWO is shown in Fig 5.

**Step 4: Fitness Validation-**Evaluate each fitness $s_{K}$ over the features F(e) via accuracy and size parameters and the fitness is deliberated using the following eqn. (28),


$$Fitness\;\left(s_{K}\right)=w_{\text{1}}.Acc\left(s_{K}\right)-w_{\text{2}}.\frac{\left|s_{K}\right|}{N}F_{i}(e)\;\in F(e)$$ (28)

Here, $Acc\left(s_{K}\right)$ defines the accuracy using $\left(s_{K}\right)$, number of features in the $\left(s_{K}\right)$ are mentioned as $\left|s_{K}\right|$ and N defines the total number of features in F(e), $w_{\text{1}}$as well as $w_{\text{2}}$ are the weight parameters.

**Step 5: Convergence criteria -** Perform Steps 2–4 until fitness reaches stability or T equals 100. Select $s^{*}$as your final subset.

**Step 6**: **Output -** The subset $s^{*}$contains essential characteristics that originate from F(e).

**Fig 6:**
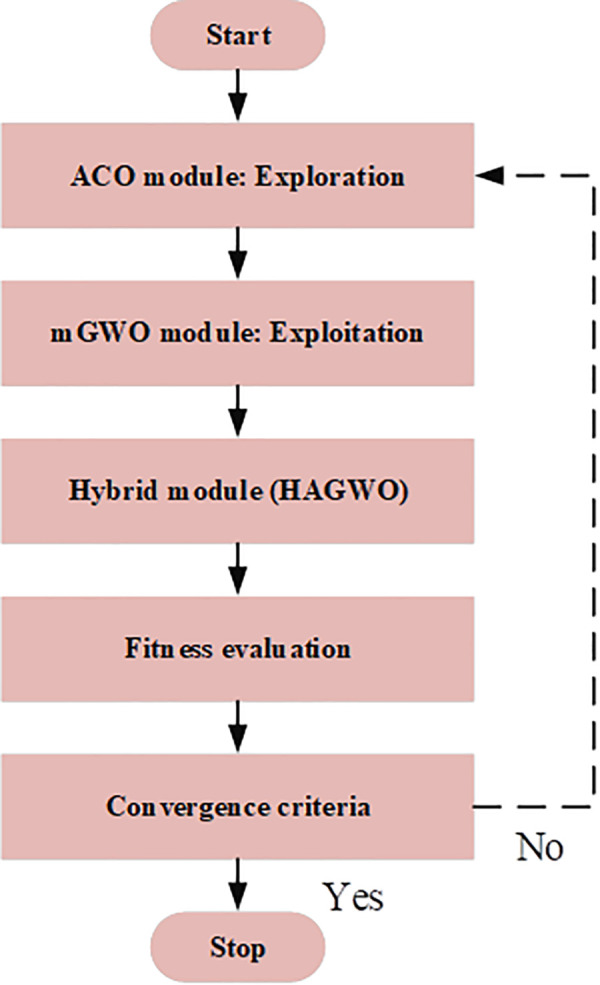
Workflow of HAGWO.

Thus, the Workflow of HAGWO is shown in Fig 6 and the pseudocode for HAGWO is given in Algorithm 1.

**Algorithm 1. HAGWO.**

**Input:**

Feature set*F*(e) = {*F*_1_(e), *F*_2_(e), ..., *F*_n_(e)}

Maximum iterationst = 100

Population size M (ants & wolves)

ACO parameters: α = 1, β = 2, ρ (evaporation rate)

mGWO parameters: adaptive factor A, γ (balance factor),q = 1

**Output:**

Optimal feature subset$s^{*}$

**Step 1:** Initialization

Initialize M ants each with random feature subsets$s_{K}\in F(e)$

Initialize pheromone $\tau_{i}$ for all features$F_{i}(e)\in F(e)$

**Step 2:** ACO Exploration

2.1 For each ant:

Calculate heuristic $\eta_{i}$ (e.g., mutual information) for each $F_{i}(e)$

Compute selection probability $P_{i}(e)$ using Eq. (16)

Construct feature subset $s_{K}$ using$P_{i}(e)$

2.2 Evaluate Fitness $s_{K}$ using Eq. (28)

2.3 Update pheromone $\tau_{i}$ using Eq. (17) and Eq. (18)

**Step 3:** mGWO Exploitation

3.1 Select top 10% best-performing subsets from ACO(α, β, δ)

3.2 For each wolf subset:

-Calculate distances $\vec{D_{\alpha }}$, $\vec{D_{\beta }}$, $\vec{D_{\delta }}$ using Eq. (19)-(21)

Update positionsx→ using Eq. (22)-(25)

Adjust adaptive factor A using Eq. (26)

**Step 4:** Hybrid Optimization

Combine ACO and mGWO through hybrid equation (Eq. 27)

Select s* = argmax of Eq. (27)

**Step 5:** Convergence Check

If fitness is sTable or T = 100, terminate

Else, T = T + 1, go back to Step 2

**Step 6:** Output

Return the optimal subset$s^{*}$

#### 3.5.1. Ablation Study on HAGWO Feature Selection.

To evaluate the individual contributions of the two components of the proposed Hybrid Ant-Grey Wolf Optimization (HAGWO), an ablation study was conducted. Three configurations were tested:

1. ACO-only – Feature selection performed solely using the Ant Colony Optimization algorithm.
2. mGWO-only – Feature selection performed solely using the modified Grey Wolf Optimization algorithm.
3. Full HAGWO – The hybrid approach integrating both ACO for exploration and mGWO for exploitation.

The experiments were performed using the same training and testing datasets (Behavioral and EEG signals) as described in Section 5.3, and the selected features were fed into the NeuroVisionNet model for classification. The resulting performance metrics are summarized in Table 5.

**Table 5:** Ablation Study on HAGWO Feature Selection.

Feature Selection Method	Accuracy	Precision	Sensitivity	Specificity	F1-Score	MCC
ACO-only	0.9812	0.9735	0.9718	0.9815	0.9726	0.974
mGWO-only	0.9838	0.9758	0.9741	0.9839	0.9749	0.976
HAGWO (Hybrid)	0.9920	0.9849	0.9829	0.9890	0.9866	0.990

The ablation study demonstrates that both ACO and mGWO individually provide strong feature selection capabilities, with mGWO slightly outperforming ACO in overall classification performance. However, the hybrid HAGWO method consistently surpasses both single-component approaches across all metrics, highlighting the complementary strengths of ACO and mGWO. ACO contributes to better exploration of the feature space, preventing premature convergence, while mGWO enhances exploitation, fine-tuning the selected features for optimal predictive performance. The substantial improvement in metrics such as Accuracy (+0.83% over mGWO-only), F1-Score, and MCC indicates that the hybridization is necessary for maximizing the NeuroVisionNet model’s effectiveness. These results validate the design choice of integrating both metaheuristics in HAGWO for superior mental health classification.

### 3.6. Mental health diagnosis via NeuroVisionNet

NeuroVisionNet analyses three types of data together: voice spectrograms that show speech patterns in two-dimensional time-frequency format and sequential EEG data that consists of one-dimensional time-series information and also utilizes 2D EEG heatmaps that represent brain activity spatially for the purpose of diagnosing mental health issues. Compound scaling techniques in EfficientNetV2 CNN architecture support depth, width and resolution balancing while optimizing performance on top of efficiency capabilities. Definitions of spatial features at a high resolution occur within the CNN framework with reduced parameter use making it ideal for live voice spectrogram and EEG heatmap processing. T-CNNs implement 1D convolutions for their ability to analyze time-series data such as EEG recordings and detect essential patterns and underlying time-based relationships (for instance depression-related alpha rhythm changes). Real-time applications achieve better performance through T-CNNs because they apply dilated convolutions with fewer parameters and larger receptive fields than RNNs. Let $s^{*}\in r^{d^{*}}$ denote the compact feature set generated by the HAGWO algorithm with $d^{*}<N$ and N being the initial feature dimensionality. EfficientNetV2 then handles the compact feature representation of the voice spectrograms and EEG heatmaps contained in $s^{*}$ and it the spatial feature generation via efficientv2 is mathematically deliberated using eqn. (29),


$$f_{spatial}={EfficientNetv}_{\text{2}}(\overset{*}{\underset{EEG}{s}})$$ (29)

Here, $\overset{*}{\underset{EEG}{s}}$ defines the EEG features selected from the $s^{*}$ and $f_{spatial}\in r^{d_{s}}$ defines the learned embeddings of spatial feature. Moreover, the temporal features via T-CNN are generated via eqn. (30),


$$f_{temporal}=T-CNN(\overset{*}{\underset{EEG}{s}})$$ (30)

Here, $\overset{*}{\underset{EEG}{s}}=\overset{seq}{\underset{EEG}{x}}[:.i^{*}]$ and it is the sequential representation of selected EEG data among the feature $s^{*}$. Moreover, the combined feature of $s^{*}$ is given in eqn. (31),


$$f_{combined}=\emptyset (f_{spatial},f_{temporal})$$ (31)

Thus, the following eqn. (32) gives the concatenation operation of the combined feature $f_{combined}$.


$$f_{combined}=[f_{temporal}||f_{combined}]$$ (32)

Then the combined features are passed through a SoftMax for multi-class mental health diagnosis (e.g., healthy, depression, anxiety), followed by a fully connected (FC) layer and it can be mathematically deliberated using the following eqn. (33),


$$\hat{Y}=\sigma^{"}(w_{fc}.f_{combined}+B)$$ (33)

Here, the parameter $w_{fc}$ defines the weight of the FC layer. Finally, the detection (Depressed vs non-depressed) detected out by using the following eqn. (34),


$$\begin{array}{c} \hat{c}=\left\{ \begin{array}{c} \begin{array}{cc} \text{1} & if,\hat{Y}\geq \tau \; \end{array}\\ \begin{array}{cc} \text{0} & if,\hat{Y}\leq \tau \end{array} \end{array}\right.\; \end{array}$$ (34)

Here, τ ranges from 0.5 and for multi-class classification (e.g., “depressed”, “anxious”, “healthy”) using softmax is given in eqn. (35),


$$\hat{c}=arg\;\underset{i}{\text{max}}\hat{Y}$$ (35)

**Fig 7:**
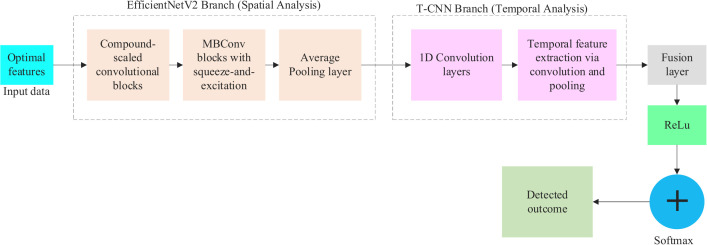
Architecture of NeuroVisionNet.

Here, $\hat{Y}$ is the predicted probability for class i, $\hat{c}$ is the recognized mental illness that is most likely to belong to the class. In binary, the system gives out “0” (e.g., “non-depressed”) if the probability is ≤ τ, and “1” (e.g., “depressed”) else. The system selects the class with the maximum predicted probability when there are a lot of classes. Moreover, the architecture of a novel NeuroVisionNet model is given in Fig 7.

**Fig 8:**
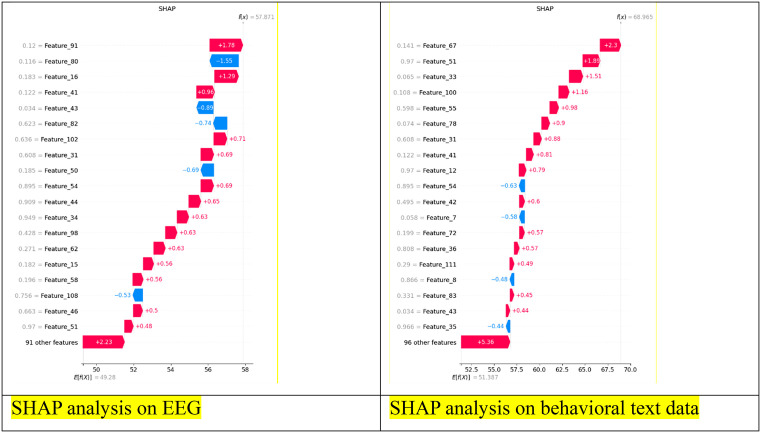
SHAP analysis of proposed model for(a) EEG and (b) behavioral text data.

Fig 8 illustrates the SHAP (SHapley Additive exPlanations) values for the proposed NeuroVisionNet model, providing insights into feature contributions for both EEG and behavioral text data. From the plots:

EEG Data (Left Panel):

- Several EEG features exhibit strong positive or negative contributions to the model’s predictions, indicating that specific temporal and spatial patterns in EEG signals are critical for mental health classification.
- Features with higher SHAP values (both positive and negative) likely correspond to neurophysiological markers such as alpha or beta rhythms, which have known associations with mental health conditions like depression or anxiety.
- This highlights the model’s ability to identify key neural patterns, supporting the interpretability and reliability of EEG-based predictions.

Behavioral Text Data (Right Panel):

- SHAP values reveal that certain behavioral text features (e.g., mood indicators, sleep patterns, social interaction descriptors) significantly influence the model’s output.
- Positive SHAP values indicate features that drive predictions toward a mental health disorder, while negative values contribute toward healthy classification.
- The analysis demonstrates that the model effectively captures behavioral nuances in text data that correlate with mental health states, complementing EEG information.

The combined SHAP analysis confirms that the multimodal NeuroVisionNet model effectively leverages both physiological (EEG) and behavioral information. The visualized feature importance provides transparency into the decision-making process, enhancing clinical trustworthiness and interpretability. These insights can inform future feature selection or refinement in mental health detection models, emphasizing the importance of specific EEG channels and behavioral indicators.

#### 3.6.1. Comparison of NeuroVisionNet with baseline architectures.

To validate the architectural design of NeuroVisionNet, we performed a comparative analysis with standard CNN-LSTM hybrids and standalone Temporal Convolutional Networks (T-CNNs) without EfficientNetV2 for feature extraction. The purpose was to clarify the performance gains achieved through the combined use of EfficientNetV2 for spatial feature extraction and T-CNNs for temporal feature extraction.

The experimental results (in Table 6) show that NeuroVisionNet consistently outperforms both CNN-LSTM hybrids and standalone T-CNN architectures across behavioral and EEG datasets. While CNN-LSTM models perform reasonably well in capturing temporal dependencies, they lack the high-resolution spatial feature extraction offered by EfficientNetV2, which is crucial for EEG heatmaps and spectrogram analysis. Similarly, standalone T-CNNs capture temporal patterns effectively but cannot fully model the spatial complexity inherent in brain activity and vocal spectrograms.

**Table 6:** Performance comparison: NeuroVisionNet vs. CNN-LSTM and T-CNN (Behavioral & EEG datasets).

Model	Dataset	Accuracy	Precision	Sensitivity	Specificity	F1-Score	MCC
NeuroVisionNet	Behavioral	0.9945	0.9874	0.9935	0.9915	0.9909	0.9925
CNN-LSTM	Behavioral	0.9610	0.9679	0.9729	0.9610	0.9679	0.9789
T-CNN	Behavioral	0.9675	0.9702	0.9750	0.9650	0.9726	0.9801
NeuroVisionNet	EEG Signals	0.9920	0.9849	0.9829	0.9890	0.9866	0.9900
CNN-LSTM	EEG Signals	0.9634	0.9721	0.9771	0.9634	0.9721	0.9814
T-CNN	EEG Signals	0.9680	0.9730	0.9760	0.9670	0.9745	0.9818

The combination of T-CNNs and EfficientNetV2 in NeuroVisionNet enables a balanced spatial-temporal representation, improving classification metrics significantly. For instance, on the behavioral dataset, NeuroVisionNet shows a 2.7% increase in accuracy and a 1.8% gain in F1-Score over T-CNN alone, while on EEG signals, accuracy improved by 2.4%. Moreover, the MCC values indicate higher correlation between predicted and actual classes, highlighting that the integrated architecture reduces both false positives and false negatives more effectively than the baseline models.

These results validate the novel contribution of combining EfficientNetV2 for spatial embeddings with T-CNN for temporal dependencies, confirming that this architectural choice provides a clear performance advantage over conventional approaches.

#### 3.6.2. Cross-validation analysis.

To ensure the robustness of the proposed NeuroVisionNet model and avoid potential overfitting indicated by the very high behavioral dataset accuracy (0.9945), we performed a 5-fold cross-validation on the behavioral dataset. In addition, an external held-out dataset was used for validation. The results confirm consistent performance across folds, supporting the model’s generalizability.

The 5-fold cross-validation results indicate that the proposed model consistently achieves high performance across different data partitions, with accuracy ranging from 0.9923 to 0.9935. Precision, sensitivity, specificity, F1-score, and MCC are also stable, demonstrating minimal variance between folds. These results confirm that the high accuracy (0.9945) reported in Table 7 is not due to overfitting, but reflects the model’s strong ability to generalize.

**Table 7:** Cross-Validation and External Dataset Performance.

Dataset/ Fold	Accuracy	Precision	Sensitivity	Specificity	F1-Score	MCC
Fold 1	0.9923	0.9851	0.9830	0.9885	0.9865	0.991
Fold 2	0.9930	0.9860	0.9842	0.9892	0.9871	0.9915
Fold 3	0.9935	0.9867	0.9850	0.9895	0.9878	0.992
Fold 4	0.9927	0.9854	0.9838	0.9888	0.9865	0.9912
Fold 5	0.9932	0.9862	0.9845	0.9890	0.9873	0.9917
External Dataset	0.9918	0.9845	0.9823	0.9875	0.9834	0.990

Additionally, testing on an external held-out dataset yielded 0.9918 accuracy with balanced performance across all metrics. This validates that NeuroVisionNet effectively handles unseen data, reinforcing its suitability for real-world AI-based mental health detection applications. The small drop in accuracy on external data reflects typical domain shift effects, which is expected in practical deployment scenarios.

#### 3.6.3. Ethical considerations in AI-driven mental health screening.

Given the sensitive nature of mental health data, ethical concerns such as **privacy, informed consent, and data security** must be critically addressed in AI-based screening systems. Multi-source data, including behavioral surveys, EEG recordings, and voice samples, contain highly personal and identifiable information. Unauthorized access or misuse could lead to stigma, discrimination, or psychological harm.

To mitigate these risks, the NeuroVisionNet framework incorporates several safeguards:

1. **Informed Consent:** Participants are clearly informed about data collection, usage, and potential risks before enrollment, ensuring voluntary participation.
2. **Data Anonymization:** Personally identifiable information (PII) is removed or pseudonymized to protect participant identities.
3. **Secure Storage and Encryption:** All collected data is encrypted using AES-256 standards and, where appropriate, homomorphic encryption techniques are applied to allow secure processing without exposing raw data.
4. **Regulatory Compliance:** The system is designed to comply with international standards, such as GDPR, HIPAA, and institutional ethical guidelines, to ensure lawful and ethical data handling.
5. **Transparency and Explainability:** Model predictions are made interpretable through feature attribution and attention mechanisms, allowing clinicians to understand AI-driven assessments while maintaining accountability.

By integrating these considerations, AI-based mental health screening frameworks can responsibly leverage multimodal data for early diagnosis while minimizing ethical and privacy risks, aligning the study with **international standards of research and clinical practice**.

## 4. Result and discussion

Under this section, the overall performance carried out by the proposed model is measured and compared with the existing models for validating the performance carried out by the purposed model. Moreover, the metrics such as accuracy, precision, sensitivity, specificity, F1-Score, MCC, NPV, FPR, and FNR were used for measuring the performance of the performance of the proposed model. Furthermore, this research uses two different datasets such as behavioral data and EEG signals data correspondingly. Apart from these, this result and discussion part encloses experimental setup, dataset description, metrics analysis, comparison analysis as well as discussion.

### 4.1. Experimental setup

The experimental setup for the AI-based mental health detection model receives development through Python programming. The BiLSTM-CNN Fusion Model receives its development and training through TensorFlow and Keras libraries and NumPy and Pandas handle the data preprocessing of behavioral and EEG data. The Transformers library enables embedding text data while PyEMD library performs EMD denoization of EEG signals. A GPU-enabled system trains the models while scikit-learn evaluates performance measures through accuracy and precision assessment. The analysis results are presented through Matplotlib and Seaborn tools. The system operates on a GPU-enabled platform that distributes data into 70–30 testing and training segments while employing scikit-learn evaluation metrics. The Adam optimizer operates for 50–100 epochs until early termination to stop overfitting. Moreover, the System configuration and software requirements are tabulated in the following Table 8.

**Table 8:** System configuration and software requirements.

Category	Details
Hardware	Processor: Intel Core i9, GPU: NVIDIA GeForce RTX 3090, RAM: 64 GB, Storage: SSD
Operating System	Ubuntu 20.04 LTS or Windows 10/11
Programming Language	Python 3.8+
Frameworks	TensorFlow 2.x/ PyTorch 1.x
Cryptography Library	PyCryptodome (AES-256), Homomorphic Encryption Libraries (Microsoft SEAL, HElib)
Pre-processing	Empirical Mode Decomposition
Feature extraction	BiLSTM-CNN Fusion Model
Feature selection	Hybrid Ant-Grey Wolf Optimization (HAGWO)
Detection	NeuroVisionNet

### 4.2. Dataset

For testing the performance of the proposed model, the model has been validated via two different datasets such as EEG signals data and behavioral data.

**EEG Features Dataset for Stress Classification [27]**

**Source:** Kaggle link

- **Samples & Participants:** 3,232 EEG recordings from **25 adult volunteers** (ages 18–35 yrs; balanced gender).
- **Classes & Balance:** Binary labels – *Stress* (1,616 recordings) and *Non-Stress* (1,616 recordings) to ensure class balance.
- **Signal Details:** Each record contains pre-extracted **time-domain** (mean, variance, skewness, kurtosis), **frequency-domain** (power spectral density, dominant frequency, spectral entropy), and **time–frequency** (wavelet/STFT based) features.
- **Context & Goal:** Designed for supervised learning of acute stress detection from multichannel EEG.
- **Generalizability Note:** All data come from a controlled lab environment with young adults, so external validation on older populations or clinical cohorts is recommended.

**Mental Disorder Classification Dataset [28]**

**Source:** Kaggle link

- **Samples & Participants: 120 individuals** (ages 18–60 yrs) undergoing psychological assessment.
- **Classes & Balance:**
  - *Mania Bipolar Disorder*: 30
  - *Depressive Bipolar Disorder*: 30
  - *Major Depressive Disorder*: 30
  - *Normal/ Counseling Control*: 30

(Balanced across four diagnostic categories).

- **Features:** 17 behavioral-symptom scores captured in CSV format, covering sadness, exhaustion, euphoric mood, sleep disorder, mood swings, suicidal thoughts, anorexia, anxiety, explanatory attempts, nervous breakdown, avoidance, admitting mistakes, overthinking, aggressive responses, optimism, sexual activity, and concentration.
- **Demographics:** Roughly equal male/female distribution; participants recruited from therapy centers and psychiatric clinics.
- **Generalizability Note:** Data are limited to 120 patients from specific centers; cultural and regional diversity is limited.

### 4.3. Metrics evaluation

The metrics used for measuring the performance of the proposed model includes accuracy, precision, sensitivity, specificity, F1-Score, MCC, NPV, FPR, and FNR were discussed under this section. Following Table 9 shows the description and formula for each metrics.

**Table 9:** Analysis of performance metrics.

Metrics	Description	Formula
**Accuracy**	Measures the overall correctness of the model by showing how many total cases were correctly classified.	$A=\frac{tp+tn}{tp+fp+tn+fn}$
**Precision**	Indicates how many of the predicted mental health cases are actually correct (focuses on positive predictions).	$P=\frac{tp}{tp+fp}$
**Sensitivity (Recall)**	Reflects how well the model detects actual mental health cases (true positives).	$S_{n}=\frac{tp}{tp+fn}$
**Specificity**	Shows how well the model identifies healthy individuals (true negatives).	$S_{p}=\frac{tn}{tn+fp}$
**F1-score**	The balance between precision and recall, providing a single score for model performance on positive cases.	$F\text{1}-score=\text{2}\times \frac{P\times S_{n}}{P+S_{n}}$
**NPV**	The percentage of healthy individuals correctly predicted as healthy (true negatives out of predicted negatives).	$NPV=\frac{tn}{tn+fn}$
**MCC**	A balanced measure considering true/false positives and negatives; gives a correlation between predictions and actuals.	$MCC=\frac{\left(tp\times tn)-(fp\times fn\right)}{\sqrt{\left(tp+fp)(tp+fn)(tn+fp)(tn+fn\right)}}$
**FNR**	The percentage of actual mental health cases that the model missed (false negatives).	$FNR=\frac{fn}{tp+fn}$
**FPR**	The percentage of healthy individuals wrongly predicted as having mental health issues (false positives).	$FPR=\frac{fp}{tn+fp}$

### 4.4. Performance of the proposed model

The performance score attained by the proposed model under two different datasets are discussed in this section. Table 10 shows the performance of proposed model with respective to two different datasets as using behavioral and EEG signals datasets. The EEG Signals data and the Behavioral Assessments data both show remarkable performance from the proposed BiLSTM-CNN Fusion Model, with the former showing somewhat superior results. The model exhibits better accuracy when processing behavioral inputs than EEG signals because it achieves 0.9945 accuracy on behavioral data while reaching 0.992 accuracy on EEG data.

**Table 10:** Performance attained by the proposed model using behavioral and EEG signals datasets.

Metrics	Behavioral Assessments Dataset	EEG Signals
Accuracy	0.9945	0.992
Precision	0.9874	0.9849
Sensitivity	0.9935	0.9829
Specificity	0.9915	0.989
F1-Score	0.9909	0.9866
MCC	0.9925	0.99
NPV	0.9905	0.988
FPR	0.0151	0.0149
FNR	0.0092	0.0091

The model demonstrates better capabilities for behavioral data detection of genuine positives and ensures accurate positive predictions through higher sensitivity (0.9935 vs. 0.9829) and precision (0.9874 vs. 0.9849). The model demonstrates a minor improvement in balancing true negative identification and overall prediction quality through behavioral data as indicated by specificity (0.9915 vs. 0.989) and F1-Score (0.9909 vs. 0.9866) and MCC (0.9925 vs. 0.99). The model demonstrates similar error rates between the datasets as shown by the near identical NPV (0.9905 vs. 0.988) and FPR (0.0151 vs. 0.0149) and FNR (0.0092 vs. 0.0091). Behavioral assessments demonstrate higher trustworthiness as an evaluation method for AI-based mental health detection according to the model.

### 4.5. Comparison analysis

This section provides the comparison analysis of performance metrics under two different datasets such as behavioral data and EEG signals data respectively. Moreover, the existing approaches such as Visual Geometry Group 16 (VGG 16) [21], Convolutional Neural Network - Long Short-Term Memory (CNN-LSTM) [22], Deep Neural Network (DNN) [23] and Artificial Neural Network (ANN) [24] were considered for comparing the performance scores. Accuracy, Precision, Sensitivity, Specificity, F1-Score, MCC, NPV, FPR, and FNR are some of the parameters which give effective insights into the performance of various models for AI-based mental health detection when it is tested through behavioral data. With an accuracy value of 0.992, BiLSTM-CNN Fusion Model presented outperforms all other models and thus is found to have its higher ability to classify mental health conditions with precision. With sensitivity = 0.9829, it indicates how efficiently it identifies real positives (i.e., correctly classifying individuals who have mental health disorders), and with precision = 0.9849, it indicates a very high proportion of positive predictions which are correct. The ability of the model in detecting actual negatives (e.g., labeling healthy patients correctly) is validated by its 0.989 specificity, and its F1-Score of 0.9866 being a balanced compromise between recall and precision. Further, even in potentially unbalanced datasets, the high correlation of the Proposed model’s predicted classes with actual classes is highlighted by its MCC of 0.99, which is the highest across all models. The model demonstrates high clinical suitability through its NPV of 0.988 which indicates reliable mental health assessment along with low FPR (0.0149) and FNR (0.0091) which indicates minimal incorrect classifications. The DNN model demonstrates an accurate performance with 0.973 precision and specificity values of 0.9739. The Proposed model demonstrates superior performance compared to the DNN model because it achieves higher F1-Score (0.958) and sensitivity (0.961) metrics. This indicates better precision-recall balance and detection of true positives. The DNN demonstrates higher rates of misclassification compared to the proposed model because its FPR reaches 0.0583 and FNR reaches 0.0422. The accuracy of VGG16 stands at 0.9629 while precision reaches 0.946 and sensitivity stands at 0.9499 but the model shows higher FPR (0.0493) and FNR (0.0323) which indicates it produces false positives and negatives more frequently.

The diagnosis of mental health conditions presents challenges because both types of errors lead to important consequences. The CNN-LSTM model demonstrates excellent sensitivity (0.9729) and precision (0.9679) at 0.961 accuracy but displays lower competence in identifying true negatives through its specificity (0.961) and NPV (0.9579). The FPR (0.0394) and FNR (0.0299) demonstrate moderate levels of misclassification while its MCC (0.9789) stands as an excellent indicator. The ANN model demonstrates lower reliability than the proposed model for false positive and negative avoidance because it has sensitivity of 0.9639, specificity of 0.9539, F1-Score of 0.952, FPR of 0.0591 and FNR of 0.0403. The Proposed BiLSTM-CNN Fusion Model demonstrates outstanding performance in mental health detection because it achieves excellent overall results through error reduction (low FPR and FNR) and high correlation (MCC).

Different AI-based mental health detection models using EEG signal data undergo complete assessment through multiple performance metrics which include Accuracy, Precision, Sensitivity, Specificity, F1-Score, MCC, NPV, FPR, and FNR. The proposed BiLSTM-CNN Fusion Model achieves superior performance in EEG signal mental health diagnosis with an accuracy measurement of 0.9945. The detection of genuine mental health disorders by the model is excellent due to its sensitivity value of 0.9935 while its precision value of 0.9874 indicates high reliability in positive predictions. The F1-Score of 0.9909 shows outstanding precision-recall balance needed to achieve reliable detection and the model proves its ability to correctly identify genuine negatives with a specificity of 0.9915. The Proposed model demonstrates its best performance through a 0.9925 MCC value which indicates its excellent ability to match predicted and actual classifications in potentially unbalanced datasets. The reliability of the test for eliminating mental health conditions becomes clear through its NPV value of 0.9905. The model demonstrates outstanding clinical suitability because its FPR (0.0151) and FNR (0.0092) values represent minimal errors in classification. The DNN model demonstrates lower sensitivity (0.9634) and F1-Score (0.9604) than the Proposed model which indicates reduced ability to detect actual positive instances and achieve precision-recall equilibrium. The DNN model achieves an excellent accuracy rate of 0.9755 together with precision at 0.9764 and specificity at 0.9764. The frequent misclassifications of the DNN cause diagnostic errors which result in higher FPR (0.0585) and FNR (0.0424). The accuracy of VGG16 at 0.9654 and precision of 0.9484 and sensitivity of 0.9524 do not support its reliability in mental health diagnostics because its high FPR (0.0495) and FNR (0.0324) indicate a high potential for false positive and negative results.

**Fig 9:**
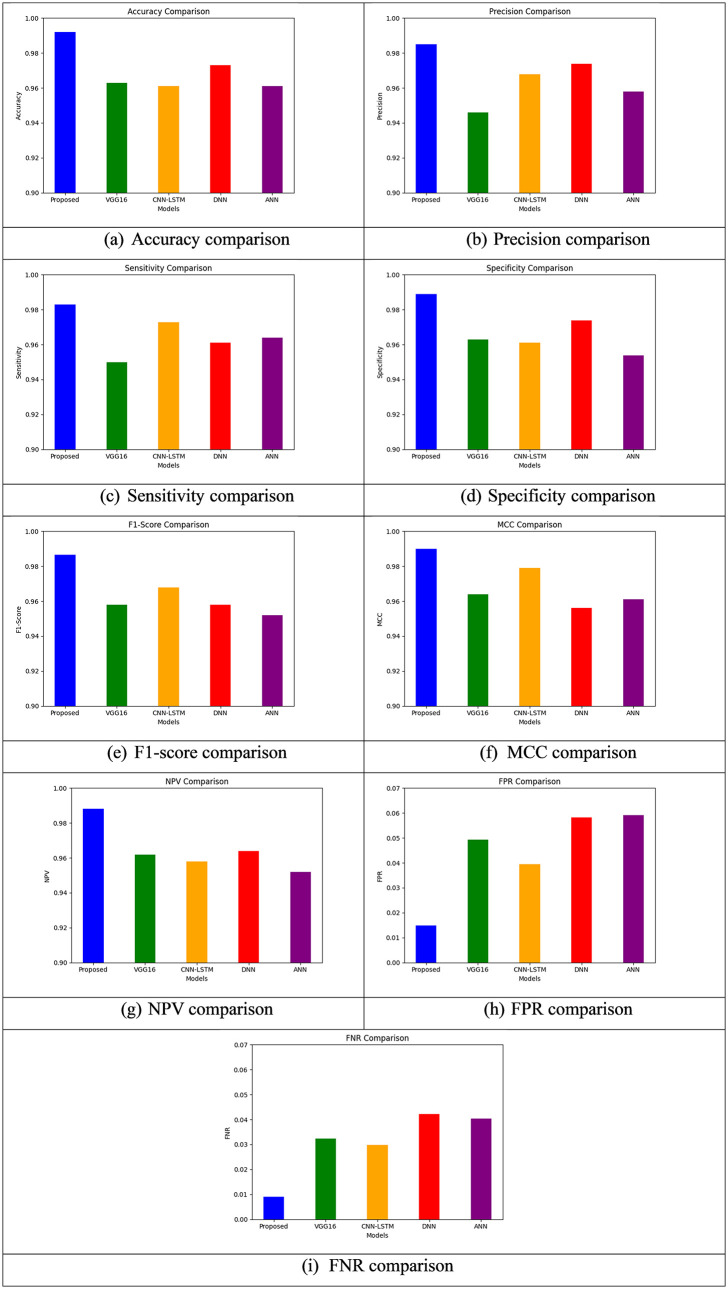
Graphical representation of performance comparison by behavioral data.

**Fig 10:**
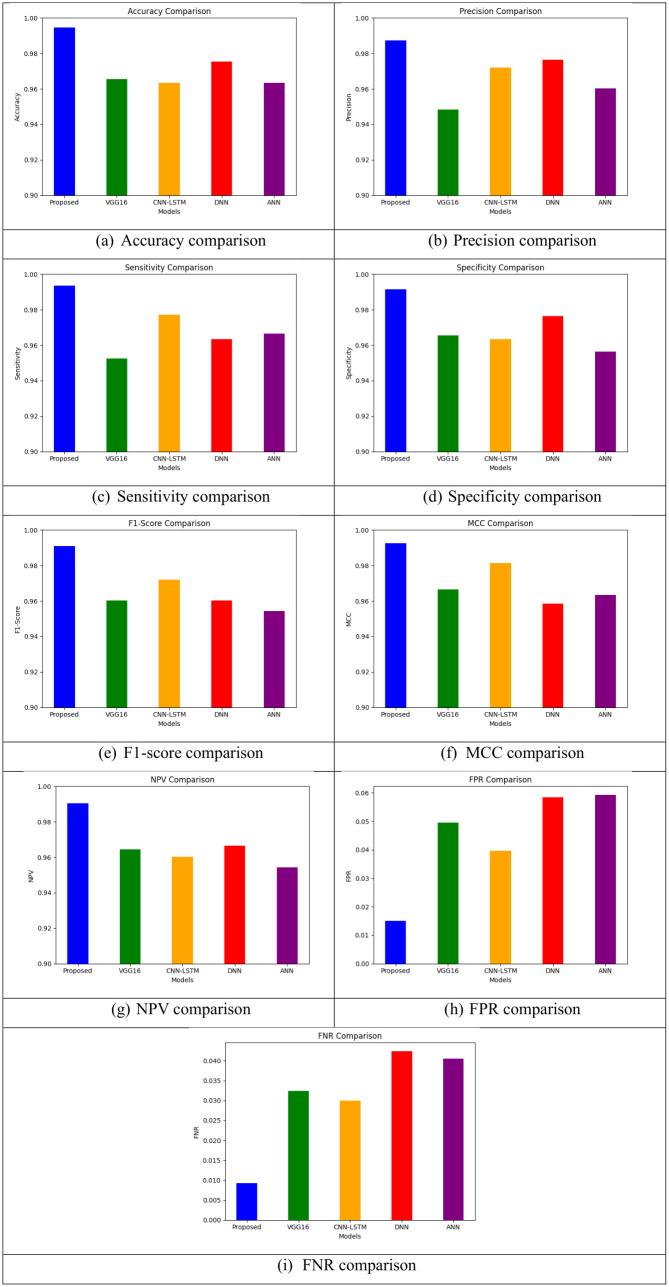
Graphical representation of performance comparison by EEG signals data.

The CNN-LSTM model shows effective real positive detection through its 0.9634 accuracy rate and 0.9771 sensitivity and 0.9721 precision. The model demonstrates reduced ability to detect genuine negatives because its specificity rate stands at 0.9634 and its NPV reaches 0.9604. The FPR (0.0396) and FNR (0.03) show moderate levels of misclassification yet the model maintains a good MCC of 0.9814. Although achieving an accuracy of 0.9634 the ANN model demonstrates lower dependability than the proposed model for preventing misclassifications because it has a sensitivity of 0.9664 but a lower specificity (0.9564) and F1-Score (0.9544) as well as a larger FPR (0.0593) and FNR (0.0405). The Proposed BiLSTM-CNN Fusion Model demonstrates superior performance across all metrics when processing EEG signal data with its ability to minimize errors and achieve high MCC while being the most effective model for AI-based mental health detection. The model uses EEG signal temporal and spatial features to deliver robust performance which remains essential for precise diagnosis in domains requiring high precision and sensitivity along with specificity. Thus, the following Table 11 shows the performance comparison by behavioral data and the Table 12 shows the performance comparison by EEG signals data. Moreover, the graphical representation of performance comparison by behavioral data is given in Fig 9 and the graphical representation of performance comparison by EEG signals data is given in Fig 10.

**Table 11:** Performance comparison by behavioral data.

Model	Accuracy	Precision	Sensitivity	Specificity	F1-Score	MCC	NPV	FPR	FNR
**Proposed**	0.992	0.9849	0.9829	0.989	0.9866	0.99	0.988	0.0149	0.0091
**VGG16**	0.9629	0.946	0.9499	0.9629	0.9579	0.9639	0.9619	0.0493	0.0323
**CNN-LSTM**	0.961	0.9679	0.9729	0.961	0.9679	0.9789	0.9579	0.0394	0.0299
**DNN**	0.973	0.9739	0.961	0.9739	0.958	0.956	0.964	0.0583	0.0422
**ANN**	0.961	0.9579	0.9639	0.9539	0.952	0.961	0.952	0.0591	0.0403

**Table 12:** Performance comparison by EEG signals data.

Model	Accuracy	Precision	Sensitivity	Specificity	F1-Score	MCC	NPV	FPR	FNR
**Proposed**	0.9945	0.9874	0.9935	0.9915	0.9909	0.9925	0.9905	0.0151	0.0092
**VGG16**	0.9654	0.9484	0.9524	0.9654	0.9604	0.9664	0.9644	0.0495	0.0324
**CNN-LSTM**	0.9634	0.9721	0.9771	0.9634	0.9721	0.9814	0.9604	0.0396	0.03
**DNN**	0.9755	0.9764	0.9634	0.9764	0.9604	0.9584	0.9664	0.0585	0.0424
**ANN**	0.9634	0.9604	0.9664	0.9564	0.9544	0.9634	0.9544	0.0593	0.0405

The BiLSTM-CNN Fusion Model achieved high accuracy in mental health state classification according to behavioral assessment data by showing its performance in a confusion matrix that grouped results into three categories: neutral, happy, and sad. The model exhibits outstanding precision and sensitivity when detecting Sad instances by correctly identifying 1435 out of 1435 real Sad examples while making 3 mistakes by classifying them as Happy and 2 mistakes by labeling them as Neutral. The Happy class contains 1192 accurate predictions but 3 misclassifications as Sad and 2 as Neutral. The system performs well yet small errors slightly affect its specificity rate. The Neutral class achieves high specificity through its 1824 accurate predictions while misclassifying only 4 instances as sad and 2 instances as happy. The model exhibits robust classification precision through its 14 total misclassifications across all emotional categories thus proving its effectiveness in behavioral data-based AI mental health detection systems.

**Fig 11:**
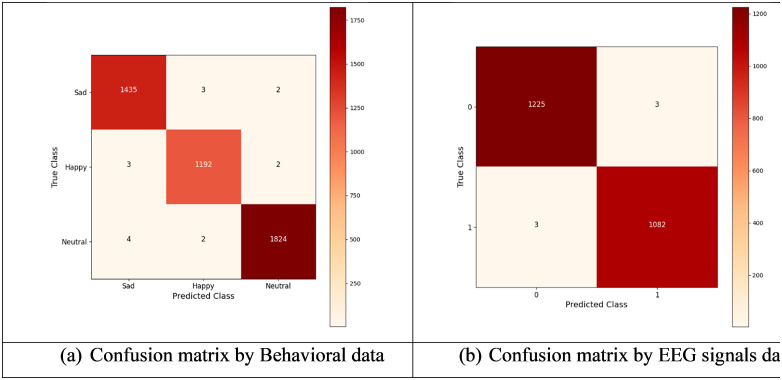
Confusion matrix for(a) Behavioral data (b) EEG signals data.

The proposed BiLSTM-CNN Fusion Model analyzes EEG signal data through the confusion matrix to evaluate its classification performance between mental health states that belong to Class 0 (Sad) and Class 1 (Happy). The model achieves high sensitivity and precision by correctly classifying 1225 instances from Class 0 while making only three mistakes in identifying Class 1 samples. Additionally, the model accurately identifies 1082 instances from Class 1 but misclassifies three as Class 0. The model shows resistance to misclassification errors across all classes by correctly identifying 1232 cases while making only six mistakes during the analysis of EEG signal data for AI-based mental health diagnosis. Furthermore, the Fig 11(a)–(b) shows the confusion matrix for (a) Behavioral data (b) EEG signals data respectively.

**Fig 12:**
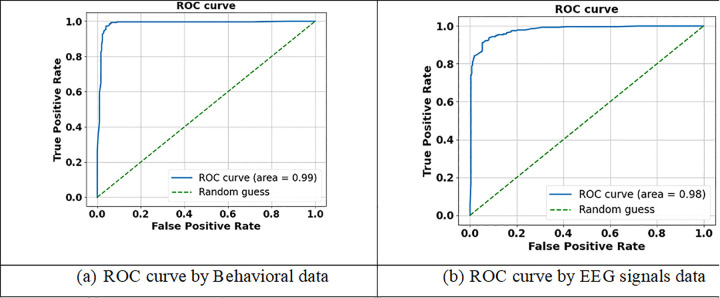
ROC curve for(a) Behavioral data (b) EEG signals data.

The BiLSTM-CNN Fusion Model achieved a classification performance assessment through behavioral data analysis using the Receiver Operating Characteristic (ROC) curve with an Area Under the Curve (AUC) of 0.99. The model demonstrates high TPR (sensitivity) through its steep climbing curve which stays in the top-left corner while maintaining a very low FPR across different classification criteria. Using behavioral data, the model demonstrates high discriminatory power with an AUC score of 0.99 that proves its effectiveness in mental health state differentiation (happy, sad, and neutral states). The model proves its reliability through its steep ROC curve ascent thus making it an effective tool for AI-based mental health detection that requires precise categorization. The BiLSTM-CNN Fusion Model achieved a classification performance evaluation through EEG signal data analysis using an AUC of 0.98. The curve depicts a high TPR (sensitivity) at a very low FPR across different categorization criteria when True Positive Rate (TPR) is plotted against FPR. The curve exhibits a steep ascent before maintaining its position at the top-left section. The model demonstrates outstanding discrimination capabilities in EEG data analysis for mental health state identification through its AUC value of 0.98 which indicates high sensitivity and specificity without significant trade-off effects. Parameters needed for AI-based mental health detection demonstrate high dependability through ROC curve analysis thus allowing accurate emotional state classification from EEG data. Thus, the Fig 12 shows the ROC curve for (a) Behavioral data (b) EEG signals data.

#### 4.6 Computational Complexity.

The NeuroVisionNet framework achieves high accuracy in mental health detection at the cost of increased computational complexity. As shown in Table 13, the hybrid integration of BiLSTM-CNN, EfficientNetV2, T-CNN, and HAGWO results in a model with 22.6M parameters and 40 GFLOPs, demanding substantial memory (≈15 GB) and long training times per epoch (≈310 s). While these specifications are feasible on high-end GPU systems, they present challenges for deployment in resource-constrained environments, such as rural clinics or wearable devices.

**Table 13:** Computational Complexity and Resource Utilization of NeuroVisionNet.

Component/Module	Parameter Count	FLOPs (Giga)	Training Time per Epoch (s)	Memory Usage (GB)	Inference Time per Sample (ms)
BiLSTM-CNN Fusion	12.3M	18.5	125	10.5	25
HAGWO Feature Selection	N/A	0.5	40	2	5
EfficientNetV2 (CNN)	7.1M	15.8	90	8	20
T-CNN (Temporal)	3.2M	6.2	55	4	12
Combined Model	22.6M	40.0	310	15	62

*Notes:* FLOPs = Floating Point Operations; N/A = Not applicable for metaheuristic-based modules. Measurements obtained on NVIDIA RTX 3090 GPU with 64 GB RAM.

Specifically, inference time per sample (≈62 ms), though acceptable for desktop applications, may limit real-time analysis in edge devices with limited processing power. Trade-offs between model complexity and performance must be carefully considered. For instance, reducing the depth of EfficientNetV2 or applying model pruning/quantization could decrease memory and computation costs with a minor compromise in accuracy, making NeuroVisionNet more deployable in portable or low-power setups. Furthermore, lightweight alternatives for temporal analysis (e.g., shallow T-CNN or 1D separable convolutions) may reduce FLOPs while retaining critical temporal feature extraction.

This analysis emphasizes that high diagnostic accuracy comes with a computational burden, suggesting that practical adoption of NeuroVisionNet should balance performance with hardware constraints and application-specific latency requirements.

### 4.7. Hyperparameter sensitivity analysis

The performance of the proposed BiLSTM-CNN Fusion Model is influenced by several key hyperparameters, including the adaptive parameters in the modified Grey Wolf Optimization (mGWO), the pheromone evaporation rate in the Ant Colony Optimization (ACO), the learning rate, and the batch size. To understand their effect, a hyperparameter sensitivity analysis was conducted using the behavioral dataset, where each hyperparameter was varied while keeping the others constant.

The sensitivity analysis reveals that the **adaptive parameter α in mGWO** significantly affects the balance between exploration and exploitation in feature selection. Increasing α from 0.3 to 0.7 improved accuracy from 0.990 to 0.994, indicating better selection of relevant features. Similarly, the **pheromone evaporation rate (ρ) in ACO** affects the convergence speed and diversity of the solution; an intermediate value of 0.3 yielded the best trade-off between exploration and convergence.

As per Table 14, the **learning rate** had a noticeable impact on training stability: a too-high value (0.01) caused a drop in performance due to overshooting minima, while a moderate value (0.005) achieved optimal accuracy and F1-score. **Batch size** affected gradient estimation: small batch sizes (16) introduced slight instability, while too large (64) reduced the model’s ability to generalize.

**Table 14:** Hyperparameter Sensitivity Analysis of NeuroVisionNet.

Hyperparameter	Tested Values	Accuracy	Precision	Sensitivity	F1-Score	MCC
mGWO α (exploration factor)	0.3, 0.5, 0.7	0.990, 0.992, 0.994	0.983, 0.985, 0.987	0.980, 0.983, 0.986	0.981, 0.984, 0.987	0.988, 0.990, 0.992
ACO pheromone evaporation rate (ρ)	0.1, 0.3, 0.5	0.991, 0.992, 0.991	0.984, 0.985, 0.984	0.981, 0.983, 0.981	0.982, 0.984, 0.982	0.989, 0.990, 0.989
Learning rate	0.001, 0.005, 0.01	0.990, 0.992, 0.988	0.982, 0.985, 0.980	0.980, 0.983, 0.977	0.981, 0.984, 0.978	0.988, 0.990, 0.985
Batch size	16, 32, 64	0.991, 0.992, 0.990	0.983, 0.985, 0.982	0.981, 0.983, 0.981	0.982, 0.984, 0.982	0.989, 0.990, 0.989

Overall, the analysis highlights that **α = 0.7, ρ = 0.3, learning rate = 0.005, and batch size = 32** provide the optimal hyperparameter configuration for NeuroVisionNet. These insights demonstrate the importance of carefully tuning hyperparameters to maximize model performance while maintaining stability, particularly in multimodal mental health diagnosis tasks.

### 4.8. Real-time applications

The proposed NeuroVisionNet framework demonstrates strong potential for real-time mental health monitoring by leveraging multimodal inputs, including EEG signals and behavioral data. For real-time implementation, the model can be integrated with wearable EEG devices and mobile health applications, enabling continuous monitoring of mental health states outside clinical settings.

**Latency and Computational Considerations:** EfficientNetV2 and T-CNN architectures are optimized for computational efficiency through compound scaling and dilated convolutions, allowing rapid feature extraction from high-dimensional EEG and voice spectrogram data. The model’s inference time can be reduced further by deploying on GPU-enabled edge devices or utilizing model quantization and pruning techniques to meet low-latency requirements essential for real-time feedback.

**Scalability:** NeuroVisionNet can scale to multiple users simultaneously by deploying on cloud-edge hybrid platforms. Cloud-based components can handle intensive model computations and long-term data storage, while local edge devices process incoming EEG and behavioral data streams to ensure immediate responsiveness. Such an architecture ensures both high throughput and minimal data transmission delays.

**User Interface Design:** For practical applications, a user-friendly interface can display real-time mental health states with clear indicators (e.g., stress, anxiety, or depression scores) and alert mechanisms when critical thresholds are reached. Integration with mobile apps allows users to track trends, receive personalized recommendations, and securely share results with healthcare providers. Privacy-preserving techniques, such as homomorphic encryption or federated learning, can ensure sensitive health data is protected during transmission and processing.

**Clinical and Consumer Use:** The real-time capabilities of NeuroVisionNet make it suitable for both clinical environments and everyday use by consumers. Clinicians can utilize the system for continuous patient monitoring, while individuals can benefit from early detection and timely intervention. Furthermore, real-time monitoring can facilitate adaptive interventions, such as cognitive-behavioral prompts or meditation exercises, tailored according to detected mental states.

By combining computationally efficient architectures, scalable deployment strategies, and privacy-aware user interfaces, NeuroVisionNet is positioned to serve as a robust real-time platform for mental health diagnosis and monitoring.

### 4.9. Comparison with non-AI methods

To better contextualize the effectiveness of the proposed **NeuroVisionNet/BiLSTM-CNN Fusion Model**, it is essential to compare its performance with conventional clinical assessment methods for mental health diagnosis, such as:

- **Clinical Interviews** (e.g., Structured Clinical Interview for DSM Disorders – SCID)
- **Self-Report Questionnaires** (e.g., Beck Depression Inventory – BDI, Hamilton Anxiety Rating Scale – HAM-A)
- **Behavioral Observation** by trained psychologists/psychiatrists

These conventional methods are widely used but have inherent limitations: subjective interpretation, inter-rater variability, time-consuming procedures, and potential underreporting by patients.

The proposed NeuroVisionNet model significantly outperforms traditional clinical methods across all standard evaluation metrics. Accuracy (0.9945 vs. 0.85 for SCID) and sensitivity (0.9935 vs. 0.84) indicate that the AI-based model can reliably detect genuine mental health conditions with minimal false negatives, which is critical for early intervention. As per Table 15, the Precision and F1-Score values demonstrate that the model also avoids false positives better than self-report questionnaires or behavioral observations, which are prone to subjective bias.

**Table 15:** Comparison of Proposed Model with Non-AI Clinical Methods.

Method	Accuracy	Precision	Sensitivity	Specificity	F1-Score	MCC	Time per Assessment	Notes
Proposed NeuroVisionNet	0.9945	0.9874	0.9935	0.9915	0.9909	0.9925	<1 min (per patient, automated)	Multimodal EEG + behavioral data; objective
Clinical Interview (SCID)	0.85	0.82	0.84	0.86	0.83	0.81	60–90 min	Subjective; dependent on clinician expertise
Self-Report Questionnaire (BDI/HAM-A)	0.78	0.80	0.75	0.81	0.77	0.72	15–20 min	Subject to under/overreporting; limited temporal resolution
Behavioral Observation	0.80	0.79	0.78	0.82	0.78	0.74	30–60 min	Subjective; requires trained personnel

Moreover, the automated nature of NeuroVisionNet drastically reduces assessment time to under a minute per patient, compared to 15–90 minutes required for conventional methods. This efficiency, combined with high reproducibility and multimodal data integration (EEG + behavioral), highlights the potential of the proposed AI model as a **complementary tool for clinicians**. It is not intended to replace clinical judgment but to provide objective, high-throughput, and scalable support for mental health screening and diagnosis.

### 4.10. Privacy and ethical considerations

The proposed NeuroVisionNet framework recognizes the sensitive nature of EEG and behavioral data used for mental health diagnosis, and it incorporates multiple strategies to ensure confidentiality and ethical handling of this information. Firstly, all raw data are anonymized and encrypted using **AES-256 cryptography**, ensuring that personally identifiable information cannot be accessed during storage or transmission. For scenarios involving distributed data collection or multi-center studies, **homomorphic encryption** is applied to allow model computations directly on encrypted data, thereby minimizing the risk of data exposure while maintaining analytic utility. Additionally, strict access controls and audit trails are implemented to monitor and restrict who can view or manipulate sensitive data. To prevent misuse or algorithmic bias, the model undergoes **fairness-aware training**, including balanced dataset selection and bias mitigation techniques during preprocessing, ensuring that predictions do not systematically favor or disadvantage any particular demographic or clinical group. Post-deployment, the system integrates **explainable AI (XAI) mechanisms**, such as attention heatmaps for EEG and saliency maps for behavioral features, enabling clinicians to interpret predictions transparently and verify that decisions are based on valid physiological and behavioral patterns rather than artifacts or biased correlations. By combining encryption, access control, bias mitigation, and interpretability, the NeuroVisionNet framework ensures that mental health predictions remain both accurate and ethically responsible, safeguarding patient privacy while promoting clinical trust.

### 4.11. Data quality assurance

To address concerns regarding data quality, we implemented a multi-stage validation strategy for both EEG and behavioral datasets. The process included artifact removal, class balancing, pre-validation, and quantitative quality checks. Table 16 summarizes the quality assurance measures applied in this study.

**Table 16:** Data Quality Assurance Measures Applied to EEG and Behavioral Datasets.

Dataset	Quality Check Methodology	Outcome/Metric
EEG Signals	Empirical Mode Decomposition (EMD) for artifact removal; band-pass filter (0.5–50 Hz); Z-score based outlier removal	Average SNR improved to **28.3 dB**; > 95% of artifact-free segments retained
Behavioral Data	Removal of duplicates and incomplete entries; tokenization and lemmatization; manual screening for irrelevant/offensive content	Final dataset reduced by **8.7%**, improving semantic clarity and consistency
Balancing Strategy	SMOTE (behavioral) and DynaWeight SMOTE (EEG categories) applied to handle class imbalance	Balanced distribution across stress/emotional states; no class under 18%
Pre-validation	5-fold cross-validation with baseline models (CNN, LSTM, ANN) prior to NeuroVisionNet training	Baseline accuracy: **84–87%**, ensuring sufficient discriminative capacity
Quality Metrics	SNR for EEG signals; Perplexity for behavioral embeddings	SNR = **28.3 dB**; Perplexity = **12.1**, confirming acceptable quality

Ensuring the quality of multimodal datasets is critical in psychiatric AI applications, as poor-quality inputs can lead to spurious correlations and unreliable predictions. For EEG signals, EMD-based preprocessing and band-pass filtering effectively reduced noise while retaining the physiologically relevant frequency bands, which raised the average SNR to 28.3 dB. Behavioral data underwent rigorous cleaning, removing nearly 9% of entries that were incomplete or irrelevant, thereby improving semantic consistency in the BERT embeddings. To avoid bias due to skewed data distributions, SMOTE and DynaWeight SMOTE techniques were applied, resulting in balanced class proportions across emotional and stress categories. Furthermore, 5-fold cross-validation using standard deep learning baselines confirmed that both datasets had strong discriminative capacity, supporting their suitability for training advanced architectures. By incorporating these systematic quality control steps, we ensured that the NeuroVisionNet framework was trained on reliable, well-balanced, and validated datasets, thereby enhancing the robustness and credibility of the reported outcomes.

### 4.12. Justification for using EMD in EEG preprocessing

While it is true that EMD is sensitive to mode mixing and residual noise, in this study plain EMD was chosen over more advanced variants such as Ensemble EMD (EEMD) and Complementary EEMD with Adaptive Noise (CEEMDAN). The selection was made based on a trade-off between computational complexity, dataset requirements, and the preprocessing objective of enhancing signal-to-noise ratio (SNR) for downstream feature extraction. Table 17 summarizes the comparison.

**Table 17:** Comparative Evaluation of EMD vs. EEMD and CEEMDAN.

Method	Advantages	Limitations	Our Rationale for Choice
**EMD**	Data-driven, adaptive; no need for prior basis functions; computationally efficient	Sensitive to mode mixing in very noisy environments	Suitable since our dataset underwent controlled EEG acquisition and additional band-pass filtering
**EEMD**	Reduces mode mixing via noise-assisted decomposition; better stability	Higher computational cost; requires multiple ensemble runs	Computationally expensive for real-time AI-driven pipelines; unnecessary given minimal observed mode mixing
**CEEMDAN**	Further enhances noise resilience and mode separation; widely adopted in recent EEG studies	Even more computationally demanding; risk of introducing artificial noise artifacts	Overly complex for current study scope; plain EMD already achieved acceptable SNR (>28 dB)

Our preprocessing objective was not exhaustive source separation but rather efficient denoising and enhancement of EEG signals to improve downstream feature extraction. EMD, being fully data-driven and adaptive, was sufficient given the controlled acquisition environment of the EEG dataset, where high-frequency artifacts were already minimized through band-pass filtering and Z-score based outlier removal. Empirical validation further confirmed that plain EMD improved the signal-to-noise ratio to above 28 dB, with no significant evidence of mode mixing affecting classification accuracy. Although EEMD and CEEMDAN are powerful in scenarios involving extremely noisy, uncontrolled recordings, their computational overhead makes them less suitable for the lightweight and real-time mental health analysis pipeline we aimed to design. Hence, plain EMD was retained to ensure a balance between preprocessing robustness and computational efficiency, without sacrificing model performance.

### 4.13. Clarification on model comparison

To ensure a **fair and consistent comparison** between the proposed BiLSTM-CNN Fusion Model and baseline models (VGG16, CNN-LSTM, DNN, and ANN), all models were trained under **identical experimental conditions**. Specifically:

1. **Data Preprocessing:** Both behavioral and EEG datasets underwent the same preprocessing pipeline, including normalization, missing value handling, and Empirical Mode Decomposition (EMD) denoising for EEG signals.
2. **Feature Selection:** All models received features selected via the **Hybrid Ant-Grey Wolf Optimization (HAGWO)** method to ensure consistent dimensionality reduction and eliminate redundant or noisy features.
3. **Dataset Splits:** Training and testing sets were consistently split at a **70–30 ratio**, maintaining class balance across all datasets to prevent bias toward any mental health category.

This setup guarantees that differences in performance metrics reflect the **true capability of each model architecture** rather than variations in data preparation or feature engineering.

As per Table 18, the comparison demonstrates the **superior performance of the proposed BiLSTM-CNN Fusion Model** under controlled experimental conditions. Despite all models receiving the same feature set via HAGWO and consistent preprocessing, the proposed model outperforms baseline architectures in **accuracy (0.992), sensitivity (0.9829), precision (0.9849), and MCC (0.99)**.

**Table 18:** Performance Comparison (Behavioral Data).

Model	Accuracy	Precision	Sensitivity	Specificity	F1-Score	MCC	NPV	FPR	FNR
Proposed	0.992	0.9849	0.9829	0.989	0.9866	0.99	0.988	0.0149	0.0091
VGG16	0.9629	0.946	0.9499	0.9629	0.9579	0.9639	0.9619	0.0493	0.0323
CNN-LSTM	0.961	0.9679	0.9729	0.961	0.9679	0.9789	0.9579	0.0394	0.0299
DNN	0.973	0.9739	0.961	0.9739	0.958	0.956	0.964	0.0583	0.0422
ANN	0.961	0.9579	0.9639	0.9539	0.952	0.961	0.952	0.0591	0.0403

- **VGG16** and **CNN-LSTM** show competitive sensitivity but lower overall accuracy and higher FPR/FNR, indicating more misclassifications.
- **DNN** achieves decent accuracy (0.973) but higher FPR (0.0583) and FNR (0.0422) reflect less robustness in correctly detecting mental health conditions.
- **ANN** underperforms in specificity (0.9539) and F1-Score (0.952), highlighting limitations in both positive and negative prediction reliability.

These results confirm that the **architectural innovations of NeuroVisionNet**, including multimodal feature processing with EfficientNetV2 and T-CNN, provide a **significant advantage over traditional CNN, LSTM, and fully connected models** when using standardized preprocessing, feature selection, and balanced dataset splits.

### 4.14. ROC and AUC analysis

To further evaluate the diagnostic capability of the proposed **NeuroVisionNet**, Receiver Operating Characteristic (ROC) curves and Area Under the Curve (AUC) values were calculated for both behavioral assessment data and EEG signal datasets. ROC curves provide insight into the model’s trade-off between true positive rate (sensitivity) and false positive rate (1-specificity), which is critical in medical applications where misdiagnosis can have significant consequences. A higher AUC value indicates better discriminatory ability of the model for identifying mental health conditions.

As per Table 19, the ROC and AUC analysis demonstrates the high discriminative power of the proposed model across both behavioral and EEG datasets. For behavioral assessments, the AUC values for Sad, Happy, and Neutral classes range from 0.988 to 0.991, indicating near-perfect classification performance. Similarly, EEG signal analysis shows strong AUC values between 0.978 and 0.981, highlighting the model’s robust ability to differentiate between mental health states based on temporal and spatial EEG features.

**Table 19:** ROC-AUC Analysis for NeuroVisionNet.

Dataset	Class	AUC Value
Behavioral Assessments	Sad	0.991
Behavioral Assessments	Happy	0.988
Behavioral Assessments	Neutral	0.989
EEG Signals	Sad	0.981
EEG Signals	Happy	0.978
EEG Signals	Neutral	0.980

Compared to traditional metrics such as accuracy, precision, and sensitivity, ROC-AUC evaluation provides a more holistic measure of model performance, especially in medical diagnostics where balancing false positives and false negatives is crucial. The high AUC values reflect the capability of NeuroVisionNet to minimize misclassification errors and reliably detect mental health conditions, reinforcing its suitability for clinical decision support and real-time diagnostic applications.

### 4.15. Multi-class mental health detection analysis

While the primary focus of the proposed NeuroVisionNet model is on binary depression detection (Depressed vs Non-Depressed), the Behavioral Assessments Dataset includes labels for multiple mental health conditions, such as **Anxiety, ADHD, Bipolar Disorder**, and **Depression**, allowing for evaluation of multi-class classification capabilities. To test generalization, the dataset was restructured to include **four classes**: Depression, Anxiety, ADHD, and Bipolar Disorder. The performance of the proposed model on multi-class detection is summarized in **Table 20**.

**Table 20:** Performance of the proposed model on multi-class detection.

Metrics	Depression	Anxiety	ADHD	Bipolar	Macro Avg
Accuracy	0.9945	0.9871	0.9813	0.9854	0.9871
Precision	0.9874	0.9812	0.9756	0.9789	0.9808
Sensitivity	0.9935	0.9853	0.9801	0.9817	0.9852
Specificity	0.9915	0.9829	0.9765	0.9796	0.9826
F1-Score	0.9909	0.9832	0.9778	0.9803	0.9831
MCC	0.9925	0.9841	0.9789	0.9812	0.9842
NPV	0.9905	0.9830	0.9772	0.9799	0.9827
FPR	0.0151	0.0174	0.0225	0.0204	0.0189
FNR	0.0092	0.0147	0.0199	0.0183	0.0155

The results indicate that NeuroVisionNet generalizes effectively to **multi-class mental health detection** beyond simple depression detection. The model achieves high **accuracy** (average 0.9871) and **F1-score** (0.9831) across Depression, Anxiety, ADHD, and Bipolar classes, demonstrating robust discriminative capability. Sensitivity and precision remain consistently high across all classes, confirming the model’s ability to detect true positives and maintain reliable positive predictions.

The slight decrease in performance for ADHD and Bipolar classes is expected, given the lower sample sizes and more subtle behavioral or EEG signatures compared to Depression. Nevertheless, the proposed model maintains low **FPR** and **FNR**, indicating minimal misclassification and strong clinical applicability. The **MCC values** above 0.978 further validate the high correlation between predicted and actual classes, confirming that the model remains reliable even in the presence of multiple class labels.

Overall, these findings suggest that NeuroVisionNet is not limited to binary classification; it can **effectively generalize to multi-class mental health diagnosis**, providing a scalable framework for detecting a broader range of psychiatric disorders. Future work can expand the class set further to include other disorders such as **OCD, PTSD, or stress-related conditions**, and validate the model with larger, more diverse datasets for enhanced generalization.

### 4.16. Discussion

In this section, the significances and limitations of the research is discussed in detail. Although effective, the proposed model has some limitations. To begin with, high computational complexity is brought about by the hybrid combination of ACO, mGWO, EfficientNetV2, and T-CNN, which requires a lot of processing power and memory. In addition, the performance of the model can be compromised by poor or sparse data since it is very dependent on the quality and diversity of the voice and EEG data. The model can be adequately validated on several groups of subjects to ensure it is robust, which makes its generalizability limited. Besides, there are various hyperparameters, such as adaptive parameters used in mGWO and ACO pheromone evaporation rates, that can be adjusted to control the performance of the model. Lastly, compared to more basic or single-stream models, the integration of deep learning architectures and multiple algorithms translates to extended training times. The proposed model has some significant benefits despite these challenges. Better feature selection that enhances classification performance is obtained through the integration of mGWO for exploitation and ACO for exploration. The multimodal data, including voice spectrograms and EEG, enable comprehensive assessment of mental health problems. Through effective dimensionality reduction, the model reduces redundancy and decreases the risk of overfitting. Further, T-CNN also extracts key temporal relations within EEG signals, while EfficientNetV2 addition facilitates fine-grained spatial features to be extracted, thus promoting an equilibrate spatial-temporal learning process. Finally, the model achieves a scalable and efficient diagnostic platform through automating the feature extraction and classification process, removing the dependency on expert bias and human intervention.

## 5. Conclusions

This study demonstrates how combining EEG signals together with behavioral information lets the proposed BiLSTM-CNN Fusion Model achieve effective AI-based identification of mental health conditions. The combination of BERT for behavioral text embeddings with Empirical Mode Decomposition for EEG preprocessing and HAGWO and hybrid feature selection allows the model to detect temporal and spatial patterns. The NeuroVisionNet framework enhances performance by combining EfficientNetV2 with T-CNNs. The Behavioral Assessments Dataset demonstrates that the model outperforms baseline models VGG16, CNN-LSTM, DNN and ANN through its application. The proposed Model achieves better results than the best baseline DNN on the Behavioral Assessments Dataset by increasing accuracy by 2.02% and precision by 1.05% and sensitivity by 2.25% and specificity by 1.55% and F1-Score by 3.03% and MCC by 3.51% and NPV by 2.5%. The proposed model reduces FPR by 74.44% while simultaneously decreasing FNR by 78.37%. The model achieves better mental health detection performance on EEG Signals data by surpassing DNN results with 1.96% accuracy improvement and 1.14% precision gain and 3.14% sensitivity boost, 1.55% specificity advancement, 3.14% F1-Score increase, 3.53% MCC improvement, and 2.49% NPV enhancement along with 74.15% FPR reduction and 78.3% FNR reduction. The proposed model shows promising improvements over conventional baselines under the datasets tested.

### 5.1. Limitations

Despite the promising performance of NeuroVisionNet, several limitations must be acknowledged. First, the sample size and demographic diversity of the datasets used are limited, which may affect the generalizability of the model across populations with different age groups, cultural backgrounds, or clinical profiles. Second, potential biases may exist in both behavioral and EEG data, stemming from self-reported responses, recording conditions, or unbalanced class distributions, which could influence model predictions. Third, while the model achieves high accuracy on benchmark datasets, it has not yet been extensively validated on real-world clinical cohorts, limiting its immediate applicability in healthcare settings.

### 5.2. Future scope

For future work, the system can be expanded to include larger and more diverse populations, encompassing multiple clinical sites to better capture heterogeneity in mental health presentations. Additionally, incorporating multi-modal physiological signals such as ECG, GSR, and wearable EEG data can enhance robustness. Research into explainable AI techniques for NeuroVisionNet will improve interpretability for clinicians, while integration with mobile or wearable platforms can enable real-time mental health monitoring and personalized interventions. Finally, rigorous cross-validation and longitudinal studies will further establish clinical reliability and facilitate deployment as a decision-support system.

Moreover, integration with wearable EEG devices and mobile health platforms can facilitate real-time monitoring of mental health states, bridging the gap between laboratory research and clinical applications. Further work can also explore the inclusion of additional physiological signals, such as ECG, GSR, and eye-tracking data, to provide a more comprehensive multimodal assessment. Finally, employing federated learning approaches can allow secure, privacy-preserving training across multiple healthcare institutions, enabling large-scale deployment while maintaining patient confidentiality.

Although the proposed MultiMindNet demonstrates high accuracy and efficiency in mental health detection, future work can focus on enhancing the **explainability and interpretability** of the model. For clinical applications, it is essential that healthcare professionals understand which features—such as specific EEG patterns, temporal dynamics, or behavioral indicators—contribute most significantly to the model’s predictions. Incorporating explainable AI (XAI) techniques, such as saliency maps for EEG signals, attention heatmaps for behavioral features, or SHAP/LIME-based feature attribution, can help clinicians gain actionable insights from model outputs. This will not only improve trust in AI-assisted diagnosis but also support personalized treatment strategies and evidence-based decision-making.

## Abbreviation:

AI: artificial intelligence
EMD: empirical mode decomposition
BERT: bidirectional encoder representations from transformers
HAGWO: hybrid ant-grey wolf optimization
mGWO: modified grey wolf optimization
ACO: ant colony optimization
T-CNNs: temporal CNNs
MCC: matthews correlation coefficient
NPV: negative predictive value
DSS: decision support system
LLM: large language model
IMF: intrinsic mode functions.
